# The Novel Role of Tyrosinase Enzymes in the Storage of Globally Significant Amounts of Carbon in Wetland Ecosystems

**DOI:** 10.1021/acs.est.2c03770

**Published:** 2022-08-09

**Authors:** Felix Panis, Annette Rompel

**Affiliations:** Universität Wien, Fakultät für Chemie, Institut für Biophysikalische Chemie, Josef-Holaubek-Platz 2, 1090 Wien, Austria

**Keywords:** climate change, global warming, peatlands, phenolics

## Abstract

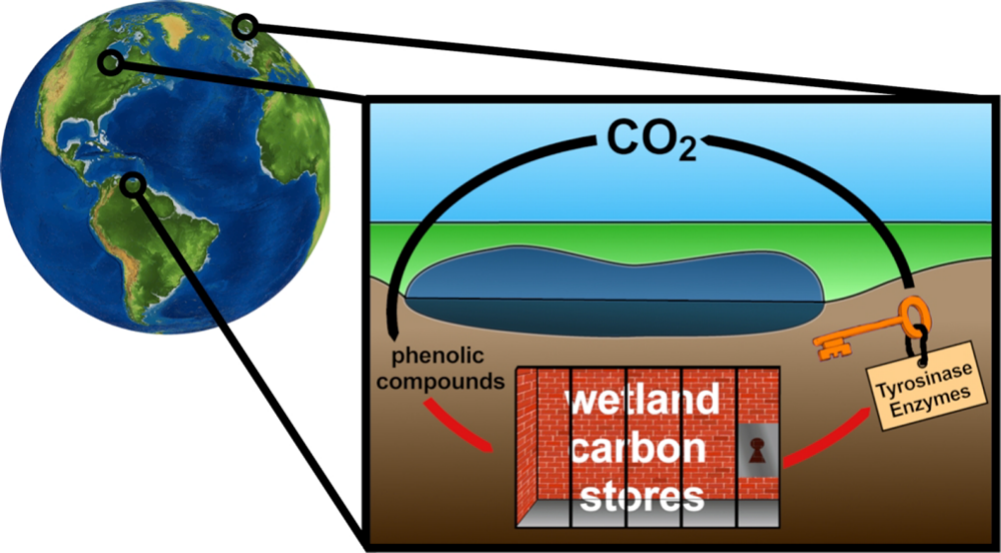

Over the last millennia, wetlands have been sequestering carbon from the atmosphere via photosynthesis at a higher rate than releasing it and, therefore, have globally accumulated 550 × 10^15^ g of carbon, which is equivalent to 73% of the atmospheric carbon pool. The accumulation of organic carbon in wetlands is effectuated by phenolic compounds, which suppress the degradation of soil organic matter by inhibiting the activity of organic-matter-degrading enzymes. The enzymatic removal of phenolic compounds by bacterial tyrosinases has historically been blocked by anoxic conditions in wetland soils, resulting from waterlogging. Bacterial tyrosinases are a subgroup of oxidoreductases that oxidatively remove phenolic compounds, coupled to the reduction of molecular oxygen to water. The biochemical properties of bacterial tyrosinases have been investigated thoroughly in vitro within recent decades, while investigations focused on carbon fluxes in wetlands on a macroscopic level have remained a thriving yet separated research area so far. In the wake of climate change, however, anoxic conditions in wetland soils are threatened by reduced rainfall and prolonged summer drought. This potentially allows tyrosinase enzymes to reduce the concentration of phenolic compounds, which in turn will increase the release of stored carbon back into the atmosphere. To offer compelling evidence for the novel concept that bacterial tyrosinases are among the key enzymes influencing carbon cycling in wetland ecosystems first, bacterial organisms indigenous to wetland ecosystems that harbor a TYR gene within their respective genome (*tyr*^+^) have been identified, which revealed a phylogenetically diverse community of *tyr*^+^ bacteria indigenous to wetlands based on genomic sequencing data. Bacterial TYR host organisms covering seven phyla (Acidobacteria, Actinobacteria, Bacteroidetes, Firmicutes, Nitrospirae, Planctomycetes, and Proteobacteria) have been identified within various wetland ecosystems (peatlands, marshes, mangrove forests, bogs, and alkaline soda lakes) which cover a climatic continuum ranging from high arctic to tropic ecosystems. Second, it is demonstrated that (in vitro) bacterial TYR activity is commonly observed at pH values characteristic for wetland ecosystems (ranging from pH 3.5 in peatlands and freshwater swamps to pH 9.0 in soda lakes and freshwater marshes) and toward phenolic compounds naturally present within wetland environments (*p*-coumaric acid, gallic acid, protocatechuic acid, *p*-hydroxybenzoic acid, caffeic acid, catechin, and epicatechin). Third, analyzing the available data confirmed that bacterial host organisms tend to exhibit in vitro growth optima at pH values similar to their respective wetland habitats. Based on these findings, it is concluded that, following increased aeration of previously anoxic wetland soils due to climate change, TYRs are among the enzymes capable of reducing the concentration of phenolic compounds present within wetland ecosystems, which will potentially destabilize vast amounts of carbon stored in these ecosystems. Finally, promising approaches to mitigate the detrimental effects of increased TYR activity in wetland ecosystems and the requirement of future investigations of the abundance and activity of TYRs in an environmental setting are presented.

## 1 Introduction

### 1.1 Classification and Biochemical Properties of Wetland Ecosystems

Wetlands are globally distributed ecosystems characterized by permanent or seasonal waterlogging, which leads to a predominantly anoxic environment.^1,2^ They cover two to six percent of the global land surface^3^ and can be divided into tidal marshes, mangrove swamps, freshwater marshes, freshwater swamps, and peatlands, depending on their structural and functional characteristics.^1^ Salt marshes are characterized by a high salinity (up to 35‰), resulting from tidal flooding. As a consequence, the vegetation of salt marshes is dominated by salt-tolerant grasses and bushes.^1^ They are distributed along coastlines in middle and high latitudes and, in tropical areas, are replaced by mangrove swamps, which also form along coastlines and exhibit a characteristic vegetation dominated by woody mangrove species.^4^ Due to evaporation, high salinity levels (>50‰) can occur in mangrove ecosystems.^5^ Tidal wetlands (salt marshes and mangrove swamps) constitute 7% of the world’s wetlands, while the remaining 93% of wetlands (freshwater marshes, freshwater swamps, and peatlands) are located inland.^6^ Freshwater marshes are, in terms of vegetation, dominated by sedges, graminoids, ferns, and herbaceous plants while freshwater swamps represent forested inland wetlands (often featuring *Alnus*, *Quercus*, *Ulmus*, *Betula*, or *Fraxinus* species).^1^ Peatlands, including bogs and fens, are located in boreal zones (83%), the tropics (13%),^7^ and the temperate zone (4%).^8^ They exhibit a characteristic vegetation of predominantly *Sphagnum* species and *Carex* species.^1,9^

Several authors reported that the microbial community of wetland ecosystems is dominated by bacteria, while fungi, archaea, and protozoa are less prevalent in terms of phylogenetic diversity and biomass.^10−13^ Proteobacteria and Actinobacteria commonly represent the dominant bacterial phyla, despite the fact that the composition of the bacterial community is dependent on various factors, such as nutrient availability, temperature, wetland type, and salinity.^10,11,13−15^ In addition, Acidobacteria, Bacteroidetes, Chloroflexi, Firmicutes, Planctomycetes, and Verrucomicrobia contribute to the bacterial community present in wetlands.^10,11,13,14^

The pH values of wetlands are influenced by various factors, such as the composition of the soil matrix, the organic matter content, the vegetation, and the microbial community.^1^ Most peatlands are characterized by acidic pH values (between pH 4 and pH 6) and show a positive correlation between acidity and organic matter content of the peat.^1^ However, depending on the flow rate and chemistry of the groundwater basic pH values have been reported for peatlands (e.g., soda lakes or rich fens), as well.^1,16^ Similar to peatlands, mangrove soils are often tilted toward mild acidity;^17,18^ however, the presence of carbonate can cause neutral or basic pH values in these ecosystems.^1,19−21^ The pH values of freshwater marshes generally range from pH 6.0 to pH 9.0,^1^ whereas freshwater swamps often show water pH values between pH 6.0 and pH 7.0. Lower pH values ranging from pH 3.5 to pH 5.0 can be observed due to the accumulation of humic acids.^1^

Wetland ecosystems characteristically exhibit high levels of phenolic compounds,^1^ which are produced by the vegetation as secondary metabolites and enter into the peatland environment either via active secretion or following cell lysis of plant litter and plant necromass.^22^ They represent a structurally diverse set of molecules including large polymers (lignin, tannins, and humic substances)^23,24^ as well as small phenolic compounds, such as flavonoids (catechin,^24^ epicatechin,^24^ epigallocatechin,^24^ isorhamnetin,^25^ kaempferol,^23,25^ quercetin,^23,25^ and taxifolin^24^), small monophenols (*p*-coumaric acid,^23,26^*p*-hydroxybenzoic acid,^26^ and sphagnum acid^27^), diphenols (protocatechuic acid,^26^ and caffeic acid^25,26^), triphenols (gallic acid^26^), and methoxylated phenols (ferulic acid,^23,26^ vanillic acid,^26^ and syringic acid^26^) (Figure S1). Due to the stabilizing resonance energy of the C–C bonds of the aromatic phenolic ring, phenolic compounds represent recalcitrant molecules.^28,29^

### 1.2 Involvement of Wetland Tyrosinases in the Global Carbon Cycle

Tyrosinases (TYRs) in wetland ecosystems, originating predominantly from bacterial species,^16^ are present in the environment due to cell lysis or following active secretion^30−32^ and are (besides other enzyme classes, including laccases and peroxidases, grouped under the generic term phenol oxidases^30,32^) capable of oxidatively lowering the concentration of phenolic compounds.^16,33^ Since TYRs require molecular oxygen they are capable of converting phenolic compounds into quinone products (for more details see section 2.1) in aerated soils, which spontaneously polymerize to form large polymers, such as melanins and humic substances.^34^ This allows TYRs to impact the global carbon cycle via three previously identified mechanisms.

First, in intact wetland ecosystems, water tables fluctuate over time,^1^ which generates an intermediate zone (located between the zones of constant waterlogging and constant aeration), in which both, oxygen and water are available. In this intermediate zone, due to increased O_2_ availability, TYRs can effectively convert small phenolic compounds into quinones,^28,29,35^ which then polymerize with other soil constituents (phenolics, amino acids, peptides and polysaccharides) to form humic substances.^36^ Humic substances are recalcitrant molecules that contribute to long-term carbon storage and stimulate plant growth.^36,37^ Thus, by contributing to the formation of humic substances, TYRs are involved in carbon storage in intact wetland ecosystems.

Second, a low molecular weight fraction of humic substances (1000–3500 g mol^–1^), termed small aquatic humic ligands (SAHLs), which are produced from phenolic precursors by TYRs (along with laccases and peroxidases) in peatlands is involved in supplying the oceans (particularly in arctic regions) with iron.^38^ Low iron concentrations (which can sink below 0.1 nmol L^–1^),^39^ caused by poor iron solubility in seawater, limit phytoplankton growth in the oceans.^40^ Complexation of iron by organic ligands, such as SAHLs, can increase its solubility by 2 orders of magnitude.^38^ Recently, it has been demonstrated that humic substances produced in peatlands collect iron (Fe(III)) by complexation and are subsequently washed away by rainfall and snowmelt. Via creeks and rivers, these humic substances–Fe(III) complexes reach the oceans, where SAHLs avoid precipitation and are transported over large distances by the ocean currents,^38^ thereby promoting phytoplankton growth, which is coupled to the sequestration of CO_2_ from the atmosphere.^41^ Human interference can severely impact the ability of TYRs present within peatlands to produce SAHLs,^42^ with far-ranging consequences for the global CO_2_ household by affecting CO_2_ sequestration in the oceans.^41^

**Figure 1:**
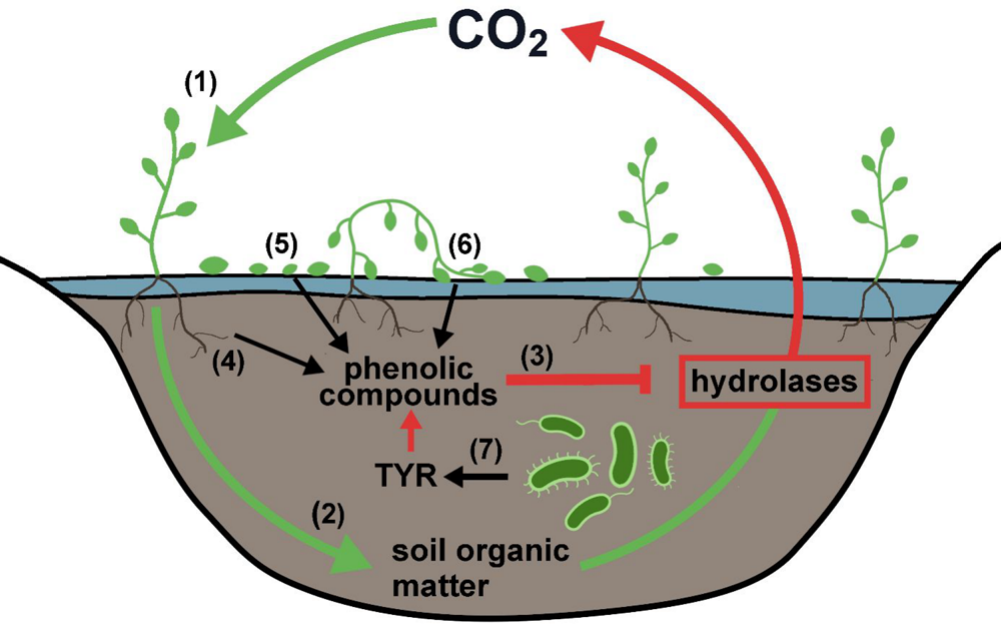
Schematic representation of the involvement of TYRs in the accumulation of organic carbon in wetland ecosystems via the “latch mechanism”.^33^ CO_2_ is converted into complex organic molecules via photosynthesis (1) and stored as soil organic matter (2), which originates predominantly from plant necromass and plant litter.^1^ The degradation of soil organic carbon via hydrolases is blocked by the high concentration of phenolic compounds (3), which are leached by the roots of wetland vegetation (4) and originate from plant litter (5) and plant necromass (6).^1,33,44^ TYRs, produced by bacteria indigenous to wetlands (7), are capable of reducing the concentration of phenolic compounds, and thus enable the degradation of soil organic matter.^33^ The figure has been edited using GIMP 2.10.18 (https://www.gimp.org).

Third, the so-called “latch mechanism” was first developed for peatlands and describes their acting as carbon sinks (Figure 1).^33^ According to this mechanism, CO_2_ is sequestered from the atmosphere via the Calvin cycle of plant photosynthesis and is stored in the form of complex organic molecules as soil organic matter, and as biomass of the peatland vegetation (which is dominated by *Sphagnum* mosses^1,22,33,43,44^). The enzymatic degradation of soil organic matter, and the subsequent release of stored organic carbon back into the atmosphere, is impeded by phenolic compounds, which are abundant in wetland ecosystems^1^ and inhibit organic matter degrading enzymes (e.g., β-glucosidases, phosphatases, xylosidases, and chitinases).^29,45−48^ The activity of phenol oxidases, such as TYRs, which are capable of reducing the concentration of inhibitory phenolic compounds, is prevented by anoxia, caused by high water tables and constant waterlogging of the catotelm,^28,29,33,35,44,49,50^ which allows phenolic compounds to accumulate in peatlands, thereby effectively suppressing the decomposition of soil organic matter. This, in turn, allows peatlands to act as net carbon sinks.^33^

Due to climate change, however, increased temperatures, reduced rainfall, and an increase in the duration and intensity of summer droughts have become likely scenarios, which will lower water tables in peatlands, thus allowing oxygen to enter into the soil. Consequently, following the oxygenation of previously anoxic peat layers, phenol oxidases, such as TYRs will be able to remove phenolic compounds. According to the “latch mechanism”, this will result in increased decomposition rates of soil organic matter and the subsequent release of vast amounts of carbon into the atmosphere, predominantly as CO_2_, which itself will promote climate change.^33^

### 1.3 Critical Assessment of the Global Impact of the “Latch Mechanism” on Carbon Storage within Wetland Ecosystems

Here, it is suggested that the “latch mechanism” can be expanded to wetland ecosystems other than peatlands, as well. Several authors reported that mangrove forests are characterized by low decomposition rates as a consequence of a high concentration of phenolic compounds and anoxia.^44,51−53^ In salt marshes, low TYR activity as a consequence of oxygen scarcity has been reported as well.^54,55^ Additionally, Rejmánková et al.^56^ investigated the concentrations of phenolic compounds in tropical and subtropical marshes, while Březinová et al.^57^ investigated phenolic compounds produced as secondary metabolites by wetland-specific vegetation in Europe. Both authors determined that high levels of phenolic compounds are present in the respective ecosystems.^56,57^ Total phenolic contents were determined using the Folin–Ciocalteu method (in which a mixture of phosphomolybdate and phosphotungstate is used for the colorimetric detection of phenolic compounds at ∼760 nm^57,58^). While this method offers information on the total concentration of phenolic compounds, it does not allow the identification of the precise chemical structure of the phenolic compounds present in the sample.^56,57^ Due to the vast prevalence of phenolic compounds in various wetland ecosystems, it is concluded that the “latch mechanism” is (among other competing and counteractive mechanisms, such as the “iron gate”^59^ and anoxic phenol metabolism^60^) involved in the stabilization of soil organic carbon on a global scale. As a consequence, wetlands represent globally significant carbon sinks, which have been sequestering CO_2_ from the atmosphere at a higher rate than releasing it for the last 4–5 millennia.^61^ Globally, wetlands are estimated to store ∼38% (550 × 10^15^ g)^62^ of the global soil organic carbon stock (1460 × 10^15^ g),^63^ which is equivalent to 73% of the amount of carbon dissolved in the atmosphere (750 × 10^15^ g), predominantly as CO_2_.^64^ So far, research investigating the “latch mechanism” in wetlands has been focused on carbon fluxes on a macroscopic scale.^65,66^ Consequently, little information is available on the specific organisms involved in the “latch mechanism”,^16^ the corresponding TYR enzymes (or other enzyme classes), their phylogenetic diversity, their biochemical properties, and their enzymatic characteristics. Here, we propose the key role of TYRs in carbon cycling in wetland ecosystems in the wake of climate change. A broad impact on human and animal wellbeing is expected. The increased emission of CO_2_ as a result of higher temperatures and reduced rainfall will profoundly influence the global CO_2_ household and, therefore, global warming.

In recent years, strong evidence supporting the single steps of the “latch mechanism” has been published. The inhibition of hydrolytic enzymes (such as β-glucosidases, phosphatases, xylosidases, and chitinases) by phenolic compounds^29,45,46,48^ and increased phenol oxidase activity as a consequence of increased O_2_ availability^28,29,33,35,44,49,50^ have been reported. Also, a reduced concentration of phenolic compounds as a consequence of increased phenol oxidase activity^28,29,33,35^ and increased activity of hydrolytic enzymes as well as an increased CO_2_ production as a consequence of a reduced concentration of phenolic compounds^28,29,33,35,44,67^ have been demonstrated. In contrast, recent studies have yielded contradictory results, as several authors reported experimental evidence questioning the inhibition of hydrolytic enzymes by phenolic compounds,^60^ increased phenol oxidase activity as a consequence of increased O_2_ availability,^68−74^ and increased activity of hydrolytic enzymes and increased CO_2_ production as a consequence of a reduced concentration of phenolics.^60^ In particular, redox reactions of amorphous iron present in wetland soils have been recognized recently as counteracting the “latch mechanism”, which has been described in detail by Wang et al.^59^ under the term “iron gate”. In short, according to the “iron gate”, the enzymatic oxidation of phenolic compounds as well as the activities of hydrolytic enzymes (with β-glucosidase often used as a model enzyme^59,68,69^) increase in the presence of Fe(II),^69,70,72,75^ which is generated in wetland soils by microbial iron reduction under anaerobic conditions.^70^ In the presence of molecular oxygen, Fe(II) is rapidly oxidized to Fe(III) oxides, which stabilize organic matter via complexation.^59,71^ The decreased activity of phenol oxidases and hydrolytic enzymes in combination with an increased stabilization of soil organic matter (both of which are effectuated by the oxidation of Fe(II) to Fe(III) following the aeration of previously anoxic wetland soils) both lead to decreased carbon mineralization rates and counteract the “latch mechanism”,^59^ which postulates increased carbon mineralization following the aeration of previously anoxic wetland soils.^33^ Recent investigations, however, refuted the “iron gate” in organic-rich wetlands.^76^ In addition, McGivern et al. identified nine enzyme groups involved in anoxic phenol metabolism by a multiomics investigation of the phenol metabolisms in a wetland in the USA which are not included under the umbrella term “phenol oxidases”.^60^

As a consequence, these contradictory results led to the conclusion that the “latch mechanism” in combination with counteractive mechanisms (such as the “iron gate”^59^ or anoxic phenol metabolism^60^) simultaneously controls carbon cycling within wetlands, with the relative potency of each mechanism depending on various factors,^77,78^ including the composition of the wetland soil,^59^ the wetland type,^79^ the wetland vegetation,^59,80^ the organic matter composition, the concentration of phenolic compounds,^68^ the duration of drought^59^ as well as the hydrological legacy,^81^ seasonal variations,^47^ the pH^80,82,83^ and the temperature of the respective wetland ecosystem,^82,83^ and the presence of enzyme inhibitors.^80^

## 2 Bacterial Tyrosinase Enzymes

### 2.1 Enzymatic Characteristics of Tyrosinases

**Figure 2:**
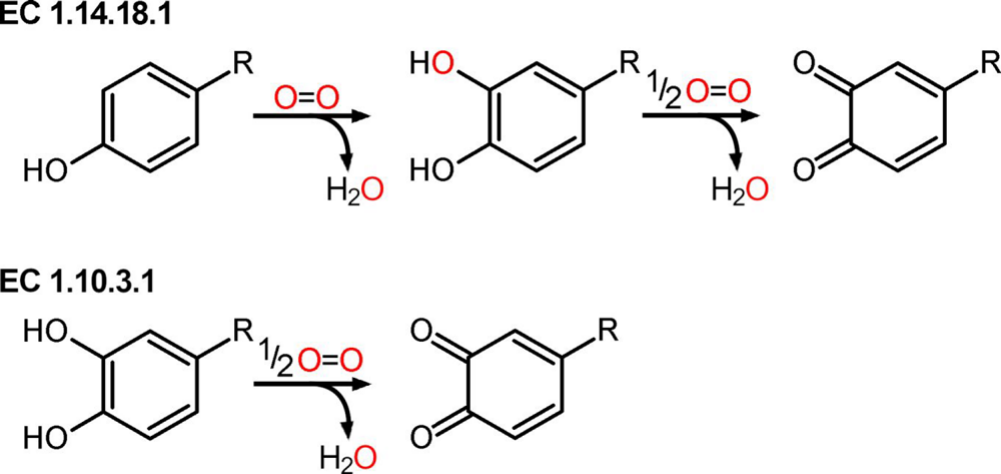
Reactions catalyzed by TYRs. Monophenols (EC 1.14.18.1) and *o*-diphenols (EC 1.10.3.1) are converted into reactive quinones by reducing molecular oxygen to water.

TYRs are oxidoreductases featuring a type III copper center, in which two copper ions are coordinated by three conserved histidine residues each^84^ (Figure S2). TYRs are bifunctional oxidoreductases, that catalyze the consecutive hydroxylation and oxidation of monophenols to *o*-quinones (EC 1.14.18.1; Figure 2 top) as well as the oxidation of *o*-diphenols to the corresponding *o*-quinones^85^ (EC 1.10.3.1; Figure 2 bottom). Accordingly, the conversion of the monophenolic substrate l-tyrosine (Figure S3) into the corresponding *o*-quinone (dopaquinone) is commonly accepted for the classification of TYRs.^34^ Reactive *o*-quinones formed by TYRs non-enzymatically polymerize.^86^

TYRs are widely distributed in nature among bacteria,^34^ archaea,^87^ fungi,^88^ plants,^89^ and animals, including humans.^90^ Melanins, formed by bacterial TYRs, are associated with radiation protection. For *Streptomyces*, it has been demonstrated that the expression of an intracellularly located TYR (referred to as “MelD”) results in a higher tolerance of the host organism toward growth-inhibiting phenolic compounds, while the presence of a second, extracellularly located TYR (referred to as “MelC”) led to the opposite effect.^91^ Consequently, the authors concluded that MelD is involved in the intracellular detoxification of phenolic compounds, while MelC is directed against competing microbes by creating growth-inhibiting *o*-quinones.^91^ In *Pseudomonas aeruginosa* a TYR enzyme (PvdP) is involved in the biosynthesis of the siderophore pyoverdine.^92^ To date, 23 TYR enzymes have been isolated from bacterial organisms and have been kinetically characterized (Table 1). Of the 23 investigated TYR enzymes, 11 have been expressed by their natural host organism while 13 TYRs have been recombinantly expressed in *E. coli* (Table 1). Phenolic compounds previously identified as TYR substrates via in vitro investigations include monophenols, diphenols, triphenols, aminophenols, nitrophenols, and halophenols (Table 1 and Figure S3). Also, flavonoids are accepted by many bacterial TYRs.

**Table 1:** Host Organisms, Investigated Substrate Scopes (Figure S3), and pH Optima of Previously Purified and Characterized Bacterial TYRs^a^

organism	PDB ID	UniProt ID	expression host	MW (kDa)	pH opt.	investigated substrate scope	ref
*Aeromonas media*	n.r.	B2Z3P7	*E. coli* BL21 (DE3), purified from natural source	57.208	pH 9.0, pH 11.0^b^	l-tyrosine (m), l-DOPA (d)	(93)
*Bacillus aryabhattai*	n.r.	A0A6H1TJ97	*E. coli* BL21 (DE3)	34.335	pH 5.0	l-tyrosine (m), l-DOPA (d)	(94)
*Bacillus megaterium*	3NM8	B2ZB02	*E. coli* BL21 (DE3)	34.410	pH 7.0	d-tyrosine (m), l-tyrosine (m), *o*-coumaric acid (m), *p*-coumaric acid (m), phenol (m), *p*-hydroxybenzoic acid (m), tyramine (m), caffeic acid (d), catechol (d), d-DOPA (d), l-DOPA (d), phloroglucin (t), pyrogallol (t), 3-aminophenol (a), catechin (f), epicatechin (f)	(95−97)
*Bacillus thuringiensis*	n.r.	n.r.	purified from natural source	∼29	pH 9.0	l-tyrosine (m), 3,4-dihydroxymandelic acid (d), 3,4-dihydroxyphenylacetic acid (d), 4-methyl catechol (d), catechol (d), dopamine (d), hydroquinone (d), l-DOPA (d), resorcinol (d)	(98)
*Burkholderia thailandensis*	5ZRE	Q2T7K1	*E. coli* BL21 (DE3)	59.024	pH 5.0	l-tyrosine (m), l-DOPA (d)	(99)
*Laceyella sacchari*	n.r.	n.r.	purified from natural source	30.910	pH 6.8	dopamine (m), l-tyrosine (m), l-DOPA (d)	(100)
*Marinomonas mediterranea*	n.r.	Q5VM57	*E. coli* BL21 (DE3)	53.040	n.r.	l-tyrosine (m), l-DOPA (d)	(101, 102)
*Pseudomonas aeruginosa*	n.r.	n.r.	*E. coli* BL21 (DE3)	61.917	n.r.	l-tyrosine (m)	(103, 104)
*Pseudomonas putida* F6	n.r.	n.r.	purified from natural source	∼36/39^d^	pH 7.0	α-methyl-dl-tyrosine (m), l-tyrosine (m), l-DOPA (d)	(105)
*Ralstonia solanacearum*	n.r.	Q8Y2J8	*E. coli* BL21 (DE3)	54.451	pH 5.0, pH 7.0^c^	l-tyrosine (m), tyramine (m), dopamine (d), l-DOPA (d), 4-bromophenol (h), 4-chlorophenol (h), 4-fluorophenol (h), 4-iodophenol (h)	(106−108)
*Rhizobium etli*	n.r.	Q8KIL0	*E. coli* strain W3110	67.418	pH 7.0	l-tyrosine (m), l-tyrosine ethyl ester (m), N-acetyl-l-tyrosine (m), caffeic acid (d), catechol (d), l-DOPA (d)	(109)
*Streptomyces albus*	n.r.	n.r.	purified from natural source	30.096	pH 7.0	l-tyrosine (m), l-DOPA (d)	(110)
*Streptomyces antibioticus*	n.r.	P07524	purified from natural source	30.739	pH 7.0	l-tyrosine (m), *p*-methylphenol (m), 4-methylcatechol (d), catechol (d), dopamine (d), epinephrine (d), hydroquinone (d), l-DOPA (d), norepinephrine (d), *p*-aminophenol (a), 4-nitrocatechol (n), *p*-nitrophenol (n), *p*-chlorophenol (h)	(111, 112)
*Streptomyces avermitilis*	6J2U	Q79ZK1	*E. coli* BL21 (DE3)	30.863	n.r.	l-tyrosine (m), l-DOPA (d), piceatannol (d), resveratrol (m,d), daidzein (f)	(113, 114)
*Streptomyces castaneoglobisporus*	2ZMZ	Q83WS2	*E. coli* BL21 (DE3)	31.039	n.r.	l-DOPA (d)	(115−117)
*Streptomyces cyaneofuscatus*	n.r.	A0A2H4QH72	purified from natural source	30.761	pH 6.5–7.5	*p*-cresol (m), caffeic acid (d), l-DOPA (d), pyrogallol (t)	(118)
*Streptomyces glaucescens*	n.r.	n.r.	purified from natural source	n.r.	pH 6.8	l-tyrosine methyl ester (m), l-DOPA (d)	(119)
*Streptomyces kathirae* SC-1	n.r.	A0A077HD11	*E. coli* JM109	30.814	pH 6.2	l-tyrosine (m), l-DOPA (d)	(120)
*Streptomyces michiganensis*	n.r.	n.r.	purified from natural source	∼32/34.5^e^	pH 7.0	α-methyl-dl-tyrosine (m), α-methyl-dl-tyrosine ethyl ester (m), l-tyrosine (m), l-tyrosine methyl ester (m), *p*-coumaric acid (m), phenol (m), caffeic acid (d), catechol (d), *L-*DOPA (d), protocatechuic acid (d)	(121)
*Streptomyces* REN-21	n.r.	n.r.	purified from natural source	∼32	pH 7.0	catechol (d), d-DOPA (d), hydroquinone (d), l-DOPA (d), catechin (f), epicatechin (f)	(122)
*Streptomyces* sp. ZL-24	n.r.	A0A2S3Y8X7	*E. coli* BL21 (DE3)	31.024	pH 9.0	l-tyrosine (m), tyramine (m), caffeic acid (d), dopamine (d), l-DOPA (d), *p*-coumaric acid (d), protocatechuic acid (d), gallic acid (t)	(16)
*Thermomicrobium roseum*	n.r.	n.r.	purified from natural source	43.000	pH 9.5	l-tyrosine (m), *o*-coumaric acid (m), *p*-coumaric acid (m), *p*-hydroxybenzoic acid (m), tyramine (m), caffeic acid (d), catechol (d), l-DOPA (d), orcin (d), resorcinol (d), phloroglucin (t), pyrogallol (t), catechin (f), epicatechin (f)	(123)
*Verrucomicrobium spinosum*	n.r.	n.r.	*E. coli* DH5α	∼ 57	n.r.	l-tyrosine (m), l-DOPA (d)	(124, 125)

a The “n.r.” (not reported) indicates that the respective parameter has not been reported.

b For the TYR from *Aeromonas media*, different pH optima have been reported for the conversion of monophenols (pH 9.0) and diphenols (pH 11.0).

c Two different TYRs were identified from *Ralstonia solanacearum*, which exhibit pH optima at pH 5.0 and pH 7.0, respectively.

d A MW of 36 kDa has been determined by size exclusion chromatography, while a MW of 39 kDa has been determined by SDS-PAGE.

e Two different bands have been identified by SDS-PAGE: (m) monophenols, (d) diphenols, (t) triphenols, (a) aminophenols, (h) halophenols, and (f) flavonoids. The chemical structure of the respective substrates is illustrated in Figures S1 and S3. pH optima were determined by photometrically measuring the conversion rates of phenolic substrates. Detailed information on the experimental setups used for the determination of the respective pH optima is reported in Table S1.

### 2.2 Structural Aspects of Bacterial TYRs

Crystal structures (PDB accession numbers: 3NM8(126) (*Bacillus megaterium*), 5ZRE(99) (*Burkholderia thailandensis*), 6J2U (*Streptomyces avermitilis*), 2ZMZ(127) (*Streptomyces castaneoglobisporus*, Table 1) of bacterial TYRs isolated from species originating from the phyla Actinobacteria,^127^ Proteobacteria,^99^ and Firmicutes^126^ have previously unveiled their structural characteristics (Figure S2). They demonstrate that the active sites of bacterial TYRs exhibit a high level of conservation, while the overall architectures of these enzymes show a surprisingly high level of variability, in contrast to plant and fungal TYRs.^128^ The six histidine residues responsible for the coordination of the two copper ions, which form the active site, are critically involved in enzymatic activity and are thus conserved in all TYR sequences identified so far.^86^ Especially conserved is the His-X-X-X-His motif (X = any amino acid), which involves the fourth (HisB1) and fifth (HisB2) Cu-coordinating histidine (Figure S2). The active site is located at the core of a four α-helical bundle, which has been observed in all structurally characterized bacterial TYRs so far,^99,126,127^ and α-helices are the main secondary structure element of bacterial TYRs. Depending on the tertiary and in some cases quaternary structural organization of bacterial TYRs, three general TYR architectures can be distinguished (Figure S4).

TYRs from *Streptomyces* species (MW ∼ 30 kDa)^34^ are expressed together with a so-called caddie protein (∼15 kDa) to form a heterodimeric TYR–caddie protein complex (Figure S4, I). The TYR harbors the active site and is responsible for the catalytic activity, while the β-sheet rich caddie protein is involved in the copper incorporation into the active site, the correct folding of the TYR, the prevention of premature activity, and in some cases the secretion of the heterodimeric complex via the TAT-secretion pathway.^129−131^ In the genomes of *Streptomyces* species, the gene coding for the caddie protein is located upstream of the gene coding for the TYR, leading to the polycistronic expression of both proteins.^132^ In vivo, TYR activity can be detected after the caddie protein detaches from its active-site-blocking location, which is effectuated by the incorporation of the two copper ions into the active site of the TYR.^129^ The high prevalence of *Streptomyces* species in soil,^133^ in combination with the fact that TYRs expressed by *Streptomyces* species are often secreted into their environment as active enzymes, makes them highly interesting enzymes in the context of soil carbon storage.

The TYRs from *Verrucomicrobium spinosum* (MW ∼ 54 kDa),^124,125^*Ralstonia solanacearum* (MW ∼ 54 kDa),^134^*Rhizobium etli* (MW ∼ 67 kDa),^135^ and *Burkholderia thailandensis* (MW ∼ 59 kDa)^99^ show a second general TYR architecture. They are produced as zymogens, which consist of an α-helix rich catalytically active domain (MW ∼ 36 kDa) harboring the dicopper site and a β-sheet rich C-terminal extension (MW ∼ 17 kDa), which adopts the role of the aforementioned caddie protein (in *Streptomyces* species) and blocks premature activity^124^ (Figure S4, II). Removal of the C-terminal domain is required to transform the zymogen-TYR (sometimes referred to as pro-TYR^124^) into its active form, which in vivo is proposed to take place by proteolytic cleavage of the C-terminal domain.^136^ Autocatalytic removal of the C-terminal domain has been reported for plant tyrosinases.^137^ A similar mechanism may be at work in bacterial TYRs; however, no experimental data regarding the in vivo activation of bacterial TYRs is available to date. In vitro, the activation process can be mimicked using SDS or trypsin as an activator.^124^ The sampling sites of *Verrucomicrobium spinosum* (freshwater lake),^124^*Rhizobium etli* (agricultural soil as a plant symbiont^109^), and *Burkholderia thailandensis* (environmental samples^99^) demonstrate that TYRs of the second general architecture are commonly present in soil samples.

The TYRs from *Bacillus megaterium* (MW ∼ 31 kDa)^95^ and *Bacillus aryabhattai* (MW ∼ 34 kDa)^138^ exemplify a third general TYR architecture. They are produced in their active forms, without a caddie protein or a C-terminal extension and are, therefore, permanently active and secreted into the environment (Figure S4, III).^136^ Since *Bacillus megaterium* and *Bacillus aryabhattai* have been isolated from soil samples,^95,138^ a possible involvement of these enzymes in soil carbons storage can be suggested, as well. Due to the natural habitats of their respective host organisms, all three general TYR architectures have been demonstrated to be present in soil and, therefore, to potentially impact soil geochemistry, in concert with other phenol oxidases.

TYRs featuring alternative architectures have been identified from *Bacillus thuringiensis* subsp. Kurstaki,^139^*Aeromonas media*,^93^ and *Marinomonas mediterranea*;^102^ however, their enzymatic classification and their in vivo existence are still under debate.^136^ The TYR of *Bacillus thuringiensis* subsp. Kurstaki represents the proteolyzed partial sequence of a full-length TYR,^136^ similar to the TYR from *Bacillus megaterium*. In contrast, the enzyme described as a TYRs identified from *Aeromonas media*(93) does not show sequence similarity to any other characterized TYRs (<15%); however, it accepts standard TYR substrates (l-tyrosine, l-DOPA, Table 1, Figure S3).^93^ Also, it does not feature the His-X-X-X-His motif,^93,102^ which is characteristic of all TYRs from plant, fungal, and bacterial sources. Thus, it is questionable if the enzyme from *Aeromonas media* represents a TYR, which exhibits a type III copper center.

### 2.3 Measurements of Tyrosinase Activity in Wetland Samples

Measuring the activities of soil samples toward phenolic compounds offers valuable information on the stability of the global soil carbon stock. However, no reliable methods for photometrically determining the ability of soils to oxidize phenolic compounds (by measuring the formation of a chromophore formed via the redox reactions catalyzed by phenol oxidases) are available yet, as reviewed previously.^32,140−142^

As an alternative to photometric assays, Walpen et al. demonstrated that a flow-injection analysis coupled to chronoamperometric detection is suitable for measuring the concentration of phenolics and quinones in peat-samples collected from an ombrotrophic bog in Sweden.^143^ In this method, the concentration of phenolic compounds is determined by measuring the reduction of ABTS^•+^ to ABTS (Figure S5), which is coupled to the oxidation of a phenol to a quinone in an oxidative cell. In parallel, the concentration of quinones is determined in a reductive cell by measuring the oxidation of ZiV^•–^ to ZiV (zwitterionic viologen, Figure S5), which is coupled to the reduction of a quinone to a phenol.^143^ This allows investigating redox processes (e.g., the oxidation of phenolic compounds to quinones by TYRs) in peat samples.^143^

Herein, we propose DNA-based investigations of the TYR community present in wetland soil samples as a promising alternative approach to the photometric quantification of the TYR activity to yield information on the presence of TYRs in wetland soils. Metagenomic sequencing projects allow to directly investigate the diversity and the composition of the microbial community capable of producing TYR enzymes. Additionally, with increasing sequence information on TYR enzymes present in wetlands, PCR-based methods, such as single-strand conformation polymorphism (SSCP) and denaturing gradient gel electrophoresis (DGGE) provide fast and cheap tools for the community analysis of TYRs present in soil samples, using TYR specific primers.^16^ Combined with microbial ecology techniques such as nucleotide analog labeling^144^ or stable isotope probing^145,146^ the effects of environmental stimuli on the subcommunity of TYR-producing bacteria can be assessed effectively. As an additional benefit, DNA-based investigations allow to selectively investigate TYRs, laccases, and peroxidases, due to specific DNA motives present in the nucleotide sequences of each of these enzyme classes. Thus, DNA based methods offer tools to closely monitor phylogenetic adaptations of TYRs (and other enzymes of interest) to environmental stresses (climate change, drought, fire, land use, freezing), which will yield valuable information on the stability of organic carbon stored in wetland soils under changing environmental conditions.

## 3 Impact of Bacterial Tyrosinase Enzymes on the Stability of Carbon Stores in Wetland Ecosystems

To impact carbon storage on a globally significant level and, by this means, influence global climate change, bacterial TYRs in wetland ecosystems must satisfy four main criteria:

I. Presence of bacteria that contain TYR genes within their genome (*tyr*^+^ bacteria) in wetland ecosystems
II. Acceptance of phenolic compounds naturally present in wetland ecosystems by tyrosinases
III. Activity of extracellular tyrosinase enzymes in their natural wetland environment
IV. Adaptation of the respective tyrosinase host organisms to their wetland environment

### 3.1 Identification of *tyr*^+^ Bacterial Species Indigenous to Wetlands

Rapid advances in the field of whole-genome sequencing and bioinformatics over the last two decades^147^ led to a plethora of fully sequenced bacterial genomes and allow one to effectively annotate protein functionalities (with 94–100% accuracy^148^) for an uncharacterized protein based on its amino acid sequence .^149,150^ The UniProt databank^151^ automatically identifies and annotates putative functionalities of uncharacterized enzymes using the InterPro database and the InterProScan software.^151^ This led to the identification and annotation of numerous TYR sequences as a byproduct of genomic sequencing projects. To identify bacterial host species indigenous to wetlands that harbor TYR genes within their respective genomes bacterial genomic sequencing data have been filtered and analyzed (see Supporting Information and Methods section 1). This resulted in the first precise report of *tyr*^+^ bacterial species identified within natural wetland ecosystems (for a detailed report of the identified TYRs, their host organisms, and their respective sampling sites please see Table S2). Artificial wetlands, such as constructed wetlands for sewage treatment and rice paddies were excluded, due to the strong interference of human activity with these ecosystems.^152^

The query terms “tyrosinase bacteria” yielded 8971 entries in the UniProt databank^153^ (August 2021), which were reviewed to identify 145 TYR enzymes (Table S3) originating from 106 bacterial species indigenous to various wetland ecosystems, including peatlands, marshes, mangrove forests, bogs, and alkaline soda lakes (Table S2). Seven bacterial phyla are presented, in alphabetical order: Acidobacteria, Actinobacteria, Bacteroidetes, Firmicutes, Nitrospirae, Planctomycetes, and Proteobacteria, including 44 different bacterial genera (Figure S6 and Table S2). The total bacterial community (*tyr*^+^ and non-TYR containing bacteria) of wetlands has previously been shown to be dominated by Proteobacteria and Actinobacteria.^15,154,155^ Similarly, most *tyr*^+^ species identified herein originate from these two phyla with 56 actinobacterial species and 38 proteobacterial species (Figure S6 and Table S2). With 35 species, *Streptomyces* (Actinobacteria) constitutes the most prominently represented genus (Table S2). The abundance of identified *Streptomyces* species (Figure S6) can be explained by the predominance of *Streptomyces* species in soil,^156^ as well as by their versatile synthetic potential, which makes them interesting research targets.^157^ Since many *Streptomyces* species express an extracellularly secreted TYR (MelC, Figure S4), they are able to oxidize phenolic compounds within their extracellular environment.^91,131,132^ Accordingly, previous studies have linked *Streptomyces* species with the storage of organic carbon in peatlands.^16,156^

An amino acid sequence alignment of all identified TYR sequences proved the presence of the conserved copper coordinating histidines (Table S3), which form the basic elements of the type III copper center. Besides these core elements, TYR sequences show a high level of diversity, ranging from 247 (Q6EH49, *Bacillus cereus*) to 701 (A0A1L3FF99, *Bradyrhizobium japonicum*) amino acids in length. Constructing a phylogenetic tree including all identified amino acid sequences of TYRs present in wetlands showed particularly strong phylogenetic clustering for actinobacterial TYR enzymes (Figure S6). Most actinobacterial TYRs (90.2%) belong to a clade exclusively featuring actinobacterial enzymes (Figure S6). Therefore, it can be assumed that these actinobacterial TYRs evolved from a common ancestor, presumably as a consequence of adaptation to diverging environmental conditions (such as substrate scope, temperature, pH) or as an adaptation to novel physiological roles. While less pronounced than for actinobacterial TYRs, also TYRs from host organisms originating from the phylum Firmicutes show some level of phylogenetic clustering. The phylogenetic tree suggests that firmicutal TYRs are most closely related to actinobacterial TYRs (Figure S6). Compared to actinobacterial and firmicutal TYRs, proteobacterial TYRs exhibit a high level of diversity. The level of sequence conservation among proteobacterial TYRs is comparable to the level of sequence conservation between Proteobacteria and different phyla (Acidobacteria, Bacteroidetes, Nitrospirae, Planctomycetes). Thus, it can be speculated that the low level of sequence conservation among proteobacterial TYRs is the consequence of a diverse set of physiological roles, leading to an equally diverse scope of substrates.

The *tyr*^+^ bacterial species indigenous to wetlands have been identified from a geographic and climatic continuum, ranging from high Arctic wetlands in Norway and Russia to tropical wetlands in Malaysia and Thailand (Figure S7 and Table S2). Zhao et al. identified a *tyr*^+^*Streptomyces* species within an alpine wetland^158^ while others reported the presence of *tyr*^+^ species in arctic regions.^159−161^ This is of particular interest since the microbial communities of wetlands located in alpine and arctic regions will be affected most severely by climate change, with potentially severe implications on the stability of organic carbon stored in these ecosystems.^162^ Out of the 106 TYR producing organisms identified within this review, 34 organisms were identified in China, followed by 22 organisms identified in India, and 19 organisms identified in Malaysia (Figure S7). So far, TYR-producing organisms indigenous to wetland ecosystems have been identified from Asia, Europe, North America, and South America, which host 87% of the global wetland area, as calculated by Davidson et al.^6^ The highest number of TYR-producing organisms indigenous to wetlands has been identified from Southeast Asia, China, and India, which can be explained by a high density of wetland ecosystems in this geographic area.^6^ In addition, based on 16S rRNA analysis it has been estimated that the total number of bacterial species present in soil samples exceeds the number of cultivatable (and therefore isolatable) species by 2 orders of magnitude.^163^ Since bacteria need to be cultured first to perform whole-genome sequencing, it becomes evident that the numbers reported herein only represent the “tip of the iceberg” and suggest a much larger, globally distributed community of TYR-producing bacteria indigenous to wetlands, yet to be identified. Nonetheless, the results presented herein prove the presence of a phylogenetically diverse community of *tyr*^+^ bacterial species present within globally distributed wetland ecosystems.

### 3.2 Acceptance of Phenolic Compounds Naturally Present in Wetland Ecosystems by Tyrosinases

Besides their mere presence, also the acceptance of phenolic compounds naturally present in wetlands by bacterial TYRs is a prerequisite for them to influence the stability of organic carbon stored within these ecosystems. Despite the fact that studies explicitly focusing on the investigation of the substrate scopes of bacterial TYRs are scarce,^95,123^ a variety of phenolic substrates accepted by bacterial TYRs has been reported so far (Table 1). The composition of phenolic compounds naturally abundant within wetland ecosystems has been investigated previously^23−27,164^ and revealed the presence of several small phenolic compounds, including benzoic acid derivatives, cinnamic acid derivatives, and flavonoids^23−27,164^ (Figures S1, S3, and S8). This allows identifying overlapping substrate scopes of characterized bacterial TYRs (Table 1) with phenolic compounds naturally present within wetland ecosystems^23−27,164^(Table 2).

**Table 2:** Phenolic Substrates Present in Wetland Ecosystems Identified as TYR Substrates^a^

substrate	wetland ecosystem	bacterial TYR
caffeic acid (Figure S1A)	mangrove swamps, peatland^25,26^	*Bacillus megaterium*(95)
		*Rhizobium etli*(109)
		*Streptomyces cyaneofuscatus*(118)
		*Streptomyces michiganensis*(121)
		*Streptomyces* sp. ZL-24^16^
		*Thermomicrobium roseum*(123)
catechin (Figure S1B)	mangrove swamps, peatland^24,27^	*Bacillus megaterium*(95)
		*Streptomyces* REN-21^122^
		*Thermomicrobium roseum*(123)
epicatechin (Figure S1C)	mangrove swamps^24^	*Bacillus megaterium*(95)
		*Streptomyces* REN-21^122^
		*Thermomicrobium roseum*(123)
gallic acid (Figure S1F)	mangrove swamps^164^	*Streptomyces* sp. ZL-24^16^
*p*-coumaric acid (Figure S1I)	peatland^23,26^	*Bacillus megaterium*(95)
		*Streptomyces michiganensis*(121)
		*Streptomyces* sp. ZL-24^16^
		*Thermomicrobium roseum*(123)
*p*-hydroxybenzoic acid (Figure S1J)	mangrove swamps^164^	*Bacillus megaterium*(95)
		*Thermomicrobium roseum*(123)
protocatechuic acid (Figure S1K)	mangrove swamps^164^	*Streptomyces michiganensis*(121)
		*Streptomyces* sp. ZL-24^16^

a “Wetland ecosystem” indicates from which type of wetland the respective substrate has been identified. “Bacterial TYR” indicates the bacterial host organisms of a TYR enzyme that have been reported to accept the respective compound as a substrate in vitro.

The following phenolic compounds are naturally present within wetland ecosystems and are in vitro accepted by bacterial TYRs as substrates: *p*-coumaric acid^23,26^ (Figure S1I), gallic acid (Figure S1F), protocatechuic acid (Figure S1K), *p*-hydroxybenzoic acid^164^ (Figure S1J), caffeic acid^25,26^ (Figure S1A), catechin^24,27^ (Figure S1B), and epicatechin^24^ (Figure S1C and Table 2). *p*-coumaric acid (Figure S1I) has been detected in a peatland,^23,26^ gallic acid (Figure S1F), protocatechuic acid (Figure S1K), *p*-hydroxybenzoic acid^164^ (Figure S1J), and epicatechin^24^ (Figure S1C) were detected in mangrove swamps, while caffeic acid^25,26^ (Figure S1A) and catechin^24,27^ (Figure S1B) were detected in peatlands and mangrove swamps (Table 2). The phenolic compound contents as well as the phenolic compound compositions of wetland ecosystems display a substantial level of variability.^23,26,164^ Tarnawaski et al. investigated the composition of two samples of medicinal peat and identified in both samples *p*-hydroxybenzoic acid as the most abundant phenolic compound, followed by protocatechuic acid and *p*-coumaric acid^26^ (Figure S1I). A study performed by Lim et al. investigated the phenolic compound composition of peat swamp samples (Malaysia) and identified flavonoids (quercetin, taxifolin, kaempferol, Figure S8) as the most abundant phenolic compounds, while *p*-coumaric acid (Figure S1I) was less prevalent.^23^ In the same study, it has been reported that the total phenolic contents show spatial and temporal variations. The highest total phenolic contents were measured at the peat surface, with significantly reduced total phenolic contents at 25 and 50 cm depth. Elevated water tables (during the wet season) also resulted in increased total phenolic contents.^23^ Moreover, in a recent study, the activity of a TYR indigenous to a peatland (*Streptomyces* sp. ZL-24, *Sz*TYR) was tested in vitro toward phenolic compounds present in its natural environment, and activity toward monophenolic, diphenolic, and triphenolic substrates was detected.^16^ Taken together, these data demonstrate that a broad spectrum of phenolic compounds naturally present in wetlands is accepted by TYRs as substrates, which substantiates a possible involvement of TYRs in the “latch mechanism” (Figure 1) as well as the formation of humic substances and SAHLs in intact peatlands. Comparing the in vitro determined kinetic parameters (*k*_cat_ and *K*_m_ values) of phenolic compounds with different functionalities toward bacterial TYRs revealed substantial variations in activity (*k*_cat_) and affinity (*K*_m_) values (Table S4). This demonstrates that different phenolic compounds potentially impact the “latch mechanism” and the formation of humic substances and SAHLs to a varying degree. Therefore, further biochemical investigations of bacterial TYRs under environmental conditions are necessary to better understand the varying impact of different phenolic compounds on global carbon cycling.

### 3.3 Potential Activity of Extracellular Tyrosinase Enzymes in Wetlands Environments

To influence the stability of soil organic carbon TYRs must display activity at ambient pH values. Despite in vitro most bacterial TYRs exhibit a pH optimum of pH 6–7, pH optima of TYRs spanning 5 pH units have been reported so far (pH 5–9, Table 1). For the TYRs from *Burkholderia thailandensis*,^99^*Ralstonia solanacearum*,^106^ and *Marinomonas mediterranea*(165,166) a pH optimum of pH 5.0 has been reported (Table 1). Notably, the TYR from *Burkholderia thailandensis* showed enzymatic activity down to a pH value of 3.0.^99^ In contrast, a pH optimum of 9.5 has been reported for the TYR from *Thermomicrobium roseum* and pH optima of 9.0 have been reported for the TYRs from *Bacillus thuringiensis*(139) and *Streptomyces* sp. ZL-24 (*Sz*TYR) (Table 1) with *Sz*TYR retaining 50% activity up to pH 11.5.^16^ When comparing the in vitro determined pH range commonly associated with TYR enzyme activity (pH 3.0^99^–11.5^16^) with the spectrum of ambient pH values characteristic for wetlands (pH 3.5–9^1^), it becomes evident that, also in terms of pH, TYR activity is compatible with wetland environments. A precise comparison of the pH optima of bacterial TYRs with ambient pH values of their sampling point would be desirable but is often impeded by a lack of reported information. Despite several previously characterized TYRs (Table 1) originate from organisms isolated from environmental samples, information on the pH value of their respective sampling site is not available in most cases: *Aeromonas media*(167) has been isolated from a lake in China; *Marinomonas mediterranea*(102) was collected from a marine environment (Mediterranean Sea); and *Bacillus megaterium*(95) (Israel), *Laceyella sacchari*(100) (Bulgaria), *Pseudomonas putida* F6,^105^*Streptomyces albus*,^110^*Streptomyces cyaneofuscatus*^118^ (Sahara desert), and *Streptomyces* REN-21^122^ (Japan) were isolated from soil samples; however, the pH values of their respective sampling sites are not reported. As an exception, *Streptomyces* sp. ZL-24^16^ was isolated from a peatland in Austria which displays a reported ambient pH value of 9.0–9.5. Interestingly, *Sz*TYR represents an extracellularly secreted enzyme and, therefore, the unusually high pH optimum of *Sz*TYR (pH 9.0) has been interpreted as a consequence of evolutionary adaptation to its natural environment.^16^

In addition to ambient pH values and the availability of substrates, the activity of extracellular enzymes in an environmental setting, including TYRs, is influenced by interactions with the soil matrix.^168^ Wetland soils can be classified as organic soil (organic matter content of >20–35%), which contain high levels of humic substances and are characteristic of northern peatlands, or mineral soils (organic matter content of <20–35%), which contain high levels of clay or sand and are often encountered in marshes and riparian forests.^1^ While TYRs have been reported to show little interaction with humic substances,^168^ they are prone to sorption to mineral surfaces (via electrostatic interactions and entropic effects), which is in general associated with an increased resistance to denaturation and proteolysis. On the other hand, interactions with clay have been reported to decrease TYR activity.^169,170^ These effects (inhibition of denaturation, proteolysis, and enzymatic activity of TYRs due to sorption to the soil matrix) can be expected to be particularly pronounced in wetland ecosystems exhibiting mineral soils. While numerous studies focused on the immobilization of TYRs for biotechnological applications,^171^ information on the sorption of TYRs in an environmental setting still represents a knowledge gap.

### 3.4 Adaptation of the Respective Tyrosinase Host Organisms to Their Wetland Environment

Besides TYRs, also their respective host organisms must show adequate levels of adaptation to their natural habitats to enable an effective production of TYR enzymes. Thus, growth conditions (pH and temperature optima) of TYR-producing bacteria indigenous to wetlands have been compared to the pH levels and climatic regions of their respective habitats. By doing this, it became evident that pH and temperature optima (in terms of growth) of bacterial TYR producing organisms indigenous to wetlands coincide with the respective ambient conditions (Table 3). Pankratov and Dedysh reported a growth optimum of 18–22 °C, with growth observed down to 2 °C for two *tyr*^+^ organisms (*Granulicella pectinivorans* and *Granulicella rosea*) identified within wetlands located in Siberia and northern Russia.^160^ Similarly, Kulichevskaya et al. reported growth down to 4 °C (with an optimum at 20–26 °C) for *Singulisphaera acidiphila*, which was identified from a northern wetland in Russia.^172^ In contrast, three *Streptomyces* species isolated from a tropical climate in Malaysia (*Streptomyces malaysiense*, *Streptomyces monashensis*, and *Streptomyces pluripotens*) exhibited a growth optimum of 28–32 °C, with no growth observed below 24 °C^173−175^ (Table 3).

**Table 3:** Adaptation of TYR-Producing Bacteria to Their Natural Habitats^a^

organism	growth (°C)	geographic location of the sampling site	mean annual temperature^176^	ref
northern regions (mean annual temperatures <5 °C)^176^				
*Granulicella pectinivorans*	2–33	wetland in North Russia	1 °C	(160)
*Granulicella rosea*	2–33	wetland in North Russia	3 °C	(160)
*Azospirillum palustre*	10–40	raised peatland in Russia	5 °C	(177)
*Methylocystis rosea*	5–37	high Arctic wetland in Norway	–10 °C	(159)
*Singulisphaera acidiphila*	4–33	northern wetland in Russia	3 °C	(172)
tropic regions (mean annual temperatures >25 °C)^176^				
*Streptomyces mangrovisoli*	24–36	mangrove forest in Malaysia	26 °C	(178)
*Streptomyces malaysiense*	26–40	mangrove forest in Malaysian	26 °C	(173)
*Streptomyces* sp. MUSC 14	26–50	mangrove forest in Malaysia	26 °C	(179)
*Streptomyces ferrugineus*	10–40	mangrove forest in Thailand	25 °C	(180)
*Streptomyces monashensis*	24–40	mangrove forest in Malaysia	26 °C	(174)
*Streptomyces pluripotens* (=MUSC 137T)	24–40	mangrove forest in Malaysia	26 °C	(175)
*Burkholderia paludis*	15–40	peatland in Malaysia	26 °C	(181)
*Rhodovulum kholense*	20–35	mangrove forest in India	25 °C	(182)

a The column “growth” indicates the temperature range (in °C) at which growth can be observed for the respective organism, as determined in an experimental setting. The column “geographic location of the sampling site” describes the global location of the natural habitat of the respective organism. Precise information on the sampling site in the form of coordinates is listed in Table S5 in the Supporting Information. The column “mean annual temperature” reports the mean annual temperature (in °C) of the sampling site of the respective organism, which demonstrates that bacterial species indigenous to arctic regions show adaptation to their natural habitat as they exhibit growth at low temperatures. In contrast, bacterial species indigenous to tropic regions exhibit growth at high temperatures, thus showing adaptation to their natural habitat. Mean annual temperatures are listed as reported by Mourshed et al.^176^

**Figure 3:**
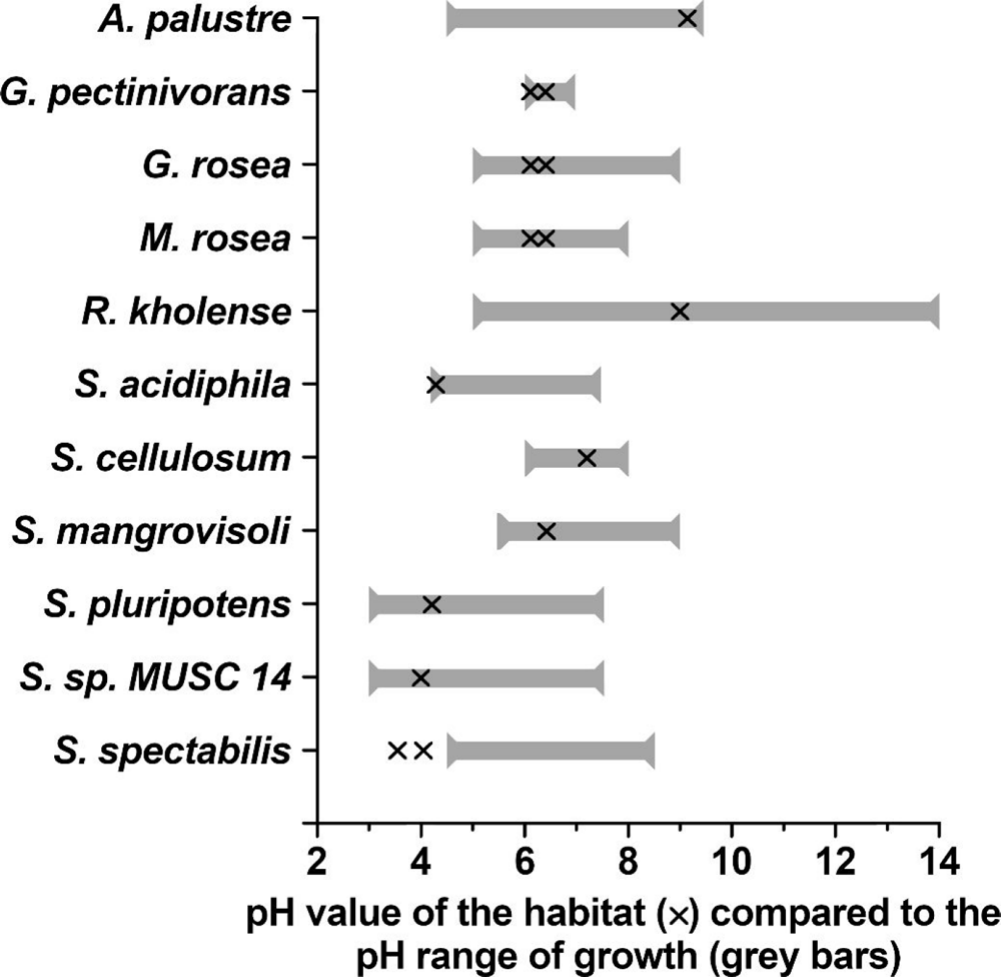
pH dependence of growth compared to ambient pH values for *tyr*^+^ bacteria indigenous to wetlands. The gray horizontal bars indicate the experimentally determined pH range at which growth of the respective organism can be observed. The black marks (×) indicate the pH of the sampling point. For some sampling points, a pH range is reported, which is represented by two marks. Organisms are ordered alphabetically. All organisms from Table S2 for which the pH value of the sampling point and a pH range at which growth can be observed are reported were included in this Figure. Detailed information on the organisms including references is presented in Table S2 in the Supporting Information. The figure has been edited using GIMP 2.10.18 (https://www.gimp.org).

Similarly, the pH value of their sampling point is well within the range at which growth can be observed in vitro for most organisms (Figure 3). Bacterial growth could be observed covering a broad pH spectrum, ranging from as low as pH 3^183^ (*Granulicella pectinivorans* and *Granulicella rosea*, Table S2) up to pH 14^184^ (*Sorangium cellulosum* So0157-2, Table S2). Interestingly, the individual ranges at which growth can be observed varied substantially for different organisms. *Sorangium cellulosum* So0157-2 exhibits a high level of euryoecious tolerance, as growth can be observed from pH 5.0 to pH 14.0^184^ (ambient pH: 9.0, Figure 3). On the contrary, for *Streptomyces* sp. MUSC 14 growth has been reported at a pH range of pH 6.0 to pH 7.0^179^ (ambient pH: 6.1–6.4, Figure 3).

### 3.5 Identification of Tyrosinases as Key Enzymes Impacting Carbon Storage in Wetland Ecosystems

Besides TYRs (EC 1.14.18.1), also laccases (EC 1.10.3.2) and peroxidases (encompassing lignin peroxidases (EC 1.11.1.14), manganese peroxidases (EC 1.11.1.13), and broad-spectrum peroxidases (EC 1.11.1.7)) represent oxidoreductases present in soils that accept a diverse scope of phenolic compounds.^32^ Peroxidases and laccases generate unstable radicals (Figure S9), which undergo a diverse set of reactions, including the polymerization and depolymerization of humic substances^185,186^ and SAHLs. Since soluble humic substances represent phenolic compounds that inhibit extracellular enzymes^187−189^ and the solubility of humic substances is negatively correlated to their molecular weight,^190,191^ the depolymerization of humic substances by laccases and peroxidases possibly leads to an ambivalent effect of these two enzyme classes on the “latch mechanisms”, as it possibly increases the solubility of phenolic compounds. Consequently, investigations of the influence of laccases on carbon cycling in peatlands led to contradictory results as increased as well as reduced CO_2_ emission rates from wetlands as a result of increased laccase activity have been reported. Zhao et al. concluded that laccases potentially increase CO_2_ emission rates from peatlands, due to the degradation of recalcitrant organic matter but on the other hand also promote carbon storage, as a result of an increased association of organic matter with iron, which is recognized as an important stabilization mechanism for soil organic matter.^192^ In contrast, quinones generated by TYRs nonenzymatically polymerize to form high-molecular-weight polymers, which are ultimately removed from the aqueous phase due to precipitation, thereby reducing their inhibitory effect on organic-matter-degrading enzymes. In addition, peroxidases and laccases are often expressed by fungal host organisms,^32,193^ while the microbial community in peatlands is dominated by bacteria.^10−13^ Also, bacteria (which often express TYRs) exhibit a roughly 10-fold higher microbial turnover, compared to fungi,^194^ which allows them to adapt faster to environmental stresses, caused by climate change. Therefore, the results reported herein support a key role of tyrosinases as regulators of organic carbon stores in wetland ecosystems. However, since no report on the phylogenetic diversity of laccases or peroxidases, their host organisms, and their global distribution in wetlands has been published so far, further investigations will be required to assess the relative impact of different enzyme classes involved in the removal of phenolic compounds (which besides tyrosinases also encompass laccases, peroxidases,^32^ and enzymes involved in anoxic phenol metabolism^60^) on the “latch mechanism” and the formation of humic substances (including SAHLs) to broaden our understanding of carbon cycling in wetland ecosystems.

## 4 Conclusions and Outlook

Herein, it is demonstrated that *tyr*^+^ bacteria have been identified within globally distributed wetland ecosystems and are well adapted to the temperature and the pH value of their respective natural habitats in terms of bacterial growth. By reviewing data collected via in vitro experiments, it is established that bacterial TYRs exhibit activity toward phenolic compounds naturally present in wetlands at ambient pH and temperature values. Thus, TYRs are proposed to act as key regulators of wetland carbon stores, together with laccases, peroxidases, and enzymes involved in anoxic phenol metabolism. To fully understand the involvement of bacterial TYRs in the storage of organic carbon in wetlands, the following research directions remain essential:

(i) Enzymatic characteristics of bacterial TYRs indigenous to peatlands (influence of pH and temperature as well as the substrate scope) in combination with their phylogenetic response to environmental stresses (heat, drought, wildfire, freezing, land use, change of vegetation due to climate change^162^) will critically influence their effects on greenhouse gas emissions.

(ii) We encourage investigations focused on the abundance and the level of expression of TYRs in a natural environment. It has been demonstrated that the expression of microbial TYRs can be induced by extracellular stimuli (such as metal ions^100,116,195^ or amino acids^100,116,196^). Since constitutive TYR expression would generate a severe energetic burden for the host organism^31^ some level of expressional control can be expected, however, factors regulating TYR expression in a natural environment still remain unclear.

(iii) Recently characterized TYRs (Table 1) have been recombinantly expressed and investigated using in vitro assays. Directly determining the effects of TYRs in an environmental setting (in contrast to in vitro experiments) will offer valuable information on their impact on the stability of carbon stored in wetlands.

To address these challenges, we propose field experiments focused on the phylogenetic abundance of TYR enzymes and *tyr*^+^ bacteria in wetlands in combination with the in vitro characterization of TYR enzymes indigenous to wetlands. Since most soil organisms (>99%) cannot be cultured readily using standard laboratory techniques,^163^ DNA-based methods and multiomic investigations will offer valuable tools for both investigations focusing on the subcommunity of *tyr*^+^ bacteria and the identification of TYR genes present in wetlands. This was underlined by a study performed by Dedysh et al. in which only 16 out of 84 bacterial 16S rRNA sequences were closely related to previously described organisms.^14^ Investigations of the subcommunity of *tyr*^+^ bacteria will elucidate the macroscopic effects of various environmental stresses (climate change, drought, fire, land use, freezing) on the activity of TYRs expressed in wetlands. This knowledge, in combination with information on additional enzyme classes involved in the wetland carbon cycle (laccases, peroxidases, and enzymes involved in anoxic phenol metabolism^60^), will be needed to improve estimations of the influence of TYRs present in wetlands on the stability of stored organic carbon and the formation of SAHLs. Moreover, the characterization of TYRs indigenous to wetlands will offer information on the precise enzymatic behaviors of these enzymes, their substrate preferences, pH- and temperature optima, and their sensitivity toward inhibitors, which should be performed under as close to environmental conditions as possible. In this context, also the identification of additional phenolic compounds naturally present in wetlands that are accepted by tyrosinases as substrates will improve our understanding of carbon cycling in wetlands. It has been reported recently that the addition of supplementary phenolic compounds to peatlands led to decreased levels of β-glucosidase activity, and subsequently to decreased levels of CO_2_ emission.^67,197^ This demonstrates that the carbon balance of wetlands can be influenced by human interventions. Over the last decades, wetlands have been receding significantly. It has been calculated that 36% of the global area covered by wetlands has been lost since 1800^198^ and in Europe, drainage and conversion to farm land alone resulted in the loss of 60% of the wetland area since 1900.^199^ Degrading peatlands alone are estimated to account for 5–10% (0.5–1.0 × 10^15^ g carbon) of the global annual anthropogenic CO_2_ emission.^200^ Thus, in recent years wetland restoration has attracted increasing attention from the scientific community and policymakers.^201^ In this context, considerable social interest can be attributed toward elucidating the role of TYRs in wetlands, as it will offer another piece of the complex puzzle that is wetland restoration. Investigating the community of *tyr*^+^ bacteria and the enzymes involved in the oxidation of phenolic compounds will offer means to develop targeted approaches for stabilizing organic carbon stored in wetlands. This will enable the mitigation of the effects of TYRs on climate change.

## Notes

A.R.: https://www.bpc.univie.ac.at/en/about-us/staff/annette-rompel/

## Notes

F.P.: https://www.bpc.univie.ac.at/en/about-us/staff/felix-panis/
