# Supplementary material for: The Novel Role of Tyrosinase Enzymes in the Storage of Globally Significant Amounts of Carbon in Wetland Ecosystems
Source: Environ Sci Technol. 2022 Aug 9;56(17):11952–68. doi: 10.1021/acs.est.2c03770 (PMC9454253; doi:10.1021/acs.est.2c03770)
Supplement: Supplementary file 1 — es2c03770_si_001.pdf [file es2c03770_si_001.pdf]

The novel role of tyrosinase enzymes in the storage of globally significant amounts of carbon in wetland ecosystems.

Felix Panis [1], Annette Rompel *[1]

1 Universität Wien, Fakultät für Chemie, Institut für Biophysikalische Chemie, Josef-Holaubek-Platz 2, 1090 Wien, Austria; https://www.bpc.univie.ac.at.

*Correspondence to: annette.rompel@univie.ac.at

Table of contents

1. Supplementary Materials and Methods 2

Methodology for the identification of putative tyrosinase sequences from genomic sequencing data. 2

2. Supplementary Tables 3

Table S1. Detailed information on the experimental setups used for the determination of pH optima of bacterial TYRs. 3

Table S2. Bacterial species indigenous to wetlands which harbor a TYR gene within their respective genome. 7

Table S3. Multiple sequence alignment. 9

Table S4. Kinetic parameters of previously characterized bacterial TYRs. 10

Table S5. Coordinates of the sampling sites of *tyr*+ bacteria listed in Table 3 of the main manuscript. 11

3. Supplementary Figures 12

Figure S1. Phenolic substrates previously identified within wetlands. 12

Figure S2. Coplot of the active centers of crystallographically characterized bacterial TYRs. 13

Figure S3. Phenolic compounds accepted as substrates by bacterial TYRs. 15

Figure S4. General architecture and cellular localization of bacterial TYRs. 16

Figure S5. Chemicals used for the chronoamperometric measurement of TYR activity. 17

Figure S6. Phylogenetic tree of the TYRs identified within the genomes of bacterial host organisms indigenous to wetland ecosystems. 18

Figure S7. Global distribution of the identified *tyr*+ organisms 19

Figure S8. Chemical structures of quercetin (A), taxifolin (B), and kaempferol (C). 20

Figure S9. Oxidation of L-DOPA by laccases, peroxidases, and tyrosinases. 21

4. References 22

1. Supplementary Materials and Methods

Methodology for the identification of putative tyrosinase sequences from genomic sequencing data. The UniProt databank was used as a source for the identification of putative TYR sequences and their respective host organisms (see UniProt identifiers in Table S2). Applying the assumption that 16S rRNA sequences exhibiting > 98.65 % nucleotide sequence identity originate from the same species[1] organisms exhibiting > 98.65 % sequence identity of 16S rRNA nucleotide sequences were defined as belonging to the same species, while organisms exhibiting < 98.65 % sequence identity of 16S rRNA nucleotide sequences were defined as belonging to different species.

All identified TYR amino acid sequences were aligned to previously enzymatically characterized and crystallized bacterial TYR amino acid sequences using the multiple sequence comparison by log-expectation (MUSCLE) algorithm implemented in the MEGA X software package (v. 10.2.1)[2] and examined for the presence of the basic structural elements (six Cu-coordinating histidines) that determine type III copper centers (Table S3).

The construction of a maximum likelihood phylogenetic tree (Figure S6) was performed by the MEGA X software package[2] using the Whelan and Goldman matrix (WAG with frequencies model implemented in the MEGA X software package) as the substitution type and by applying gamma distribution. The phylogenetic tree (Figure S6) was edited using GIMP 2.10.18 (https://www.gimp.org).

2. Supplementary Tables

Organism	Substrate	Buffer	Temp.	Ref.
Aeromonas media	*L*-DOPA	acetate buffer pH 4.0–5.0, PBS buffer pH 5.0–7.0, Tris–HCl buffer pH 7.0–9.0, glycine-NaOH buffer pH 8.5–10.5, CAPS buffer pH 10.0–12.0	n.r.	3
Bacillus aryabhattai	*L*-tyrosine	Tris-HCl pH 3.0–9.0	n.r.	4
Bacillus megaterium	*L*-tyrosine/*L*-DOPA	acetate buffer pH 4.0–5.0, potassium phosphate buffer pH 6.0–7.0, glycine-NaOH buffer pH 8.0–10.0	25 °C	5
Bacillus thuringiensis		glycine-NaOH buffer pH 8.5–10.5	42 °C	6
Burkholderia thailandensis	*L*-tyrosine	n.r.	n.r.	7
Laceyella sacchari	n.r.	n.r.	n.r.	
Marinomonas mediterranea	n.r.	n.r.	n.r.	
Pseudomonas aeruginosa	n.r.	n.r.	n.r.	
Pseudomonas putida F6	*L*-tyrosine/*L*-DOPA	sodium phosphate buffer (pH: n.r.)	30 °C	8
Ralstonia solanacearum	*L*-tyrosine/*L*-DOPA	phosphate buffer (pH: n.r.)	n.r.	9
Rhizobium etli	*L*-DOPA	acetate buffer, phosphate buffer, Tris–HCl buffer (pH: n.r)	30 °C	10
Streptomyces albus	n.r.	Davies-buffer pH 4–10	n.r.	11
Streptomyces antibioticus	*L*-DOPA	Davies-buffer (pH: n.r)	22 °C	12,13
Streptomyces avermitilis	n.r.	n.r.	n.r.	
Streptomyces castaneoglobisporus	n.r.	n.r.	n.r.	
Streptomyces cyaneofuscatus	*L*-DOPA	acetate buffer pH 3–5.5, potassium phosphate buffer pH 6–7.5, Tris–HCl buffer pH 8–10	n.r.	14
Streptomyces glaucescens	*L*-tyrosine methyl ester/*L*-DOPA	n.r.	n.r.	15
Streptomyces kathirae SC-1	*L*-DOPA	acetate buffer pH 3.0–6.0, phosphate buffer pH 6.0–8.0, Tris-HCl pH 9.0	30 °C	16
Streptomyces michiganensis	*L*-DOPA	sodium phosphate buffer (pH: n.r.)	28 °C	17
Streptomyces REN-21	*L*-DOPA	n.r.	n.r.	18
Streptomyces sp. ZL-24	tyramine	sodium phosphate buffer pH 5.5−7.5, Tris−HCl buffer pH 7.5−9.5, CAPS buffer pH 9.5−11.5	25 °C	19
Thermomicrobium roseum	*L*-DOPA	Tris−HCl (pH: n.r)	50 °C	20
Verrucomicrobium spinosum	n.r.	n.r.	n.r.	

Table S1. Detailed information on the experimental setups used for the determination of pH optima of bacterial TYRs. “Substrate” indicates which substrate has been used and “Buffer” indicates which buffers have been used to evaluate the respective pH optimum. “Temp.” indicates at which temperature the measurements were performed. “n.r.” (not reported) indicates that the respective parameter has not been reported. PBS = phosphate

buffered saline; CAPS = N-cyclohexyl-3-aminopropanesulfonic acid; TRIS = tris(hydroxymethyl)aminomethane. Chemical structures of substrates are illustrated in Figure S3.

**Organism (number of species)**	**UniProt ID**	**Sampling site**	**Ref.**
**Acidobacteria (4)**			
Acidobacteria bacterium	A0A3N5X831	peatland in Russia	21,22
Candidatus sulfopaludibacter sp. SbA4	A0A2U3KIR3<br>A0A2U3LIS9	acidic peatland in Germany	23–25
Granulicella pectinivorans	A0A1I6M5T1	sphagnum peat in Russia	26,27
Granulicella rosea	A0A239DL37	sphagnum peat in Russia	27
**Actinobacteria (54)**			
Actinobacteria bacterium	A0A3N5LM39	wetlands in the USA	21
Agromyces brachium	A0A6I3M9Z3	mangrove rhizosphere in Japan	28
Gordonia sp. HNM0687	A0A6L7GYJ8	mangrove soil in China	29
Mycolicibacterium fluoranthenivorans	A0A1G4V5R8	mangrove soil in China	30
Mycolicibacterium moriokaense	A0A370D666	mangroves in China	31
Pseudonocardia endophytica	A0A4V2PI10	mangrove ecosystem in India	32
Sphaerisporangium krabiense	A0A7W9DMW4	mangrove forest in Thailand	33
Streptomyces albus	A0A0B5F6T3<br>A0A0B5EPG7	peat soil in Russia	34
Streptomyces angustmyceticus	A0A5J4L7U6	mangrove soil in China	30
Streptomyces antibioticus	A0A1S9P1Z7<br>A0A1S9NU67	mangrove soil in China	30,35
Streptomyces aurantiacus JA 4570	S3ZAM0<br>S3ZGJ1	mangrove ecosystem in India	36
Streptomyces badius	A0A328J9V5	mangrove soil in China	37
Streptomyces caelestis	A0A0M8QIS7<br>A0A7W9H3D1<br>A0A7W9HDB9	mangrove swamp in India	38
Streptomyces candidus	A0A7X0HLM5<br>A0A7X0HJ80	peat	39
Streptomyces chattanoogensis	A0A0N0H3U4	mangrove soil in China	30
Streptomyces clavuligerus	B5GYQ0<br>B5H1U8	mangrove sediment in India	40
Streptomyces coelicoflavus	A0A6N9UN96	mangrove soil in India	41
Streptomyces colonosanans	A0A1S2Q1S7	mangrove forest in Malaysia	42
Streptomyces corchorusii	A0A124HPH4	peat forest in Malaysia	43
Streptomyces diastaticus	A0A7K3Q557	mangrove sediment sample from China	44
Streptomyces ferrugineus	A0A7M2SPF7<br>A0A7M2SBB5	mangrove soil in Thailand	45
Streptomyces gardneri	A0A4Y3S0B0<br>A0A4Y3RCW8	mangrove soil in China	37
Streptomyces globisporus	A0A0U3KNS5	peat forest in Malaysia<br>mangroves in India	40,43
Streptomyces griseoruber	A0A101SXR6<br>A0A101SWH6	mangrove swamp in India	38
Streptomyces griseus	A0A2X2M3Y0	mangroves in China	46,47

	A0A2X2M5T2		
Streptomyces libani subsp. Libani	A0A640TR07	mangroves in China	31
Streptomyces lipmanii	A0A285D923	mangrove soil in China	37
Streptomyces lydicus	A0A0D4DDN2	mangrove swamp in India	48
Streptomyces malaysiense	A0A1J4PWI9	mangrove forest in Malaysia	49
Streptomyces mangrovisoli	A0A1J4NW81<br>A0A1J4P798	mangrove forest in Malaysia	50
Streptomyces monashensis	A0A1S2PQK3<br>A0A1S2PKP4	mangrove soil in Malaysia	51
Streptomyces nigra	A0A2S1IMI8<br>A0A2S1ICI5	mangrove soil in China	52
Streptomyces olivaceus	A0A1D8SP36	mangrove ecosystem in China;<br>mangrove ecosystem in India	31,53
Streptomyces olivoverticillatus	A0A7W7LLS9	mangrove swamp in India	38
Streptomyces pactum	A0A1S6JCQ5	mangrove ecosystem in China	31
Streptomyces paludis	A0A345I2D0	alpine wetland in China	54
Streptomyces parvulus	A0A191UXZ3	peat from Malaysia;<br>mangrove soil in China	30,31,43
Streptomyces parvus	A0A5M8GTV4	mangrove soil in China	37
Streptomyces pluripotens (=MUSC 137T)	A0A221NXB2<br>A0A221P6L7	mangrove soil in Malaysia	55
Streptomyces qinzhouensis	A0A5B8IB42<br>A0A5B8JHY3	mangrove soil in China	56
Streptomyces rochei	A0A510D900<br>A0A6I5MM61	mangrove sediment in India	40
Streptomyces roseus	A0A0J6XJT5<br>A0A0J7AI25	peat soils in Russia	34
Streptomyces showdoensis	A0A2P2GLL6<br>A0A2P2GJB2	mangrove swamp in India	38
Streptomyces sp. MUSC 14	A0A1S2PK94<br>A0A1S2P7Y5	mangrove forest in Malaysia	57–59
Streptomyces sp. PCS3-D2	A0A024YX16<br>A0A024YX26	mangrove soil in the Philippines	60
Streptomyces sp. S816	A0A4V3S5K9	mangrove soil in the Philippines	61
Streptomyces sp. ZL-24	A0A2S3Y8X7<br>A0A2S3XTZ2	wetland in China;<br>soda lake in Austria	19,62
Streptomyces spectabilis	A0A516RAC2<br>A0A516R853	drained peat soils in Russia;<br>wetland in China	34,63
Streptomyces tanashiensis	Q9F1K7	mangrove soil in China	37
Streptomyces venezuelae	F2RIZ9<br>F2RLH9	mangrove swamp in India	38
Streptomyces violaceoruber	A0A1V0UKR2<br>A0A1V0UF35	peat	39
Streptomyces virginiae	A0A0L8M4B5<br>A0A0L8M4C5	mangrove soil in China	30
Streptomyces viridochromogenes	D9XAS3<br>D9X674	peat	39
Streptomyces wuyuanensis	A0A1H0A5D2<br>A0A1H0C516	mangrove soil in China	30
**Bacteroidetes (1)**			
Chryseobacterium scophthalmum	A0A1N6FBL6	wetland in India	64
**Firmicutes (8)**			
Bacillus alvei	A0A383RI28	mangrove sediment in Indonesia	65
Bacillus cereus	Q6EH49	mangroves in Malaysia;<br>mangrove peat in Brazil;<br>mangrove soil in India; mangrove	64,66–69

		swamps in Thailand; wetland in India	
Bacillus megaterium	D5DHA8	mangrove soil in India	70,71
Bacillus thuringiensis	A0A0F6J033	mangrove forest in India; mangrove soil in Saudi Arabia; mangrove soil in Japan	72–74
Bacillus toyonensis	A0A2C5KJN2	mangrove sediment in Indonesia	75
Brevibacillus laterosporus GI-9	H0UHZ0	suburban wetland in China	76
Paenibacillus polymyxa	A0A1D7MFL7<br>A0A1D7MCL4	peatland in Germany	77
Priestia aryabhattai	A0A6H1TJ97	mangroves in China	31
**Nitrospirae (1)**			
Nitrospira defluvii	D8PI13	peatland after a wildfire in Russia	78
**Planctomycetes (2)**			
Frigoriglobus tundricola	A0A6M5YW59	littoral tundra wetland in Russia	79
Singulisphaera acidiphila	L0D705	northern wetland in Russia	80–82
**Proteobacteria (36)**			
Acinetobacter guillouiae	A0A6A1RQV4	mangrove soil in India	83
Azospirillum lipoferum	A0A1X7NGR1<br>G7Z6L5	raised *Sphagnum* bogs in Russia	84
Azospirillum oryzae	A0A6N1APE8	raised *Sphagnum* bogs in Russia	84
Azospirillum palustre	A0A2B8BFB1	raised peatland in Russia	85
Azospirillum sp. Sh1	A0A5A9EZ76	raised *Sphagnum* bogs in Russia	84
Bradyrhizobium japonicum	A0A1L3FP25<br>A0A1L3FF99<br>A0A1L3FQJ7	primary deep peat in Malaysia	43
Breoghania sp. L-A4	A0A346R2L6<br>A0A346R3B5<br>A0A346QXY8	costal wetland in China	86
Burkholderia gladioli	A0A2A7SC70	mangrove sediments in Brazil	87
Burkholderia paludism	A0A6J5CVK2	tropical peat swamp soil in Malaysia	82,88
Caballeronia sp. SBC1	A0A6G8NL20	peat from the eastern European tundra	89
Chromobacterium sphagni	A0A1S1WVR1<br>A0A1S1WVB3	*Sphagnum* bog in the USA	90
Chromobacterium vaccinii	A0A1D9LLW6<br>A0A1D9LEY9	wild cranberry bog in the USA	82,91
Deltaproteobacteria bacterium	A0A538QY07	wetland in the USA	21
Erythrobacter mangrove	A0A7D3Y0J0	mangrove soil in China	92
Hahella sp. CCB-MM4	A0A261GRE4<br>A0A261GVB1	mangrove forest in Malaysia	93,94
Janthinobacterium lividum	A0A7H8RK67	wetland in India	64
Melittangium boletus DSM 14713	A0A250IQN3	no information available	82
Mesorhizobium loti	A0A1B4YH80	primary deep peat from Malaysia	43
Methylobacter tundripaludum	A0A2S6H7D7	high arctic wetland in Norway	95
Methylocystis rosea	A0A6B8LR17	high arctic wetland in Norway	82,95,96
Nitrospirillum iridis	A0A7X0B1W6	wetland in Korea	82,97
Paludibacterium purpuratum	A0A4R7BDA0	wetland in Korea	82,98
Pseudomonas fluorescens	J2Y1W2	mangrove soil in Hong Kong; mangrove soil in India; wetland in China	99–101
Pseudomonas koreensis	A0A4U3F4Z1	wetland in India	64
Pseudomonas simiae	A0A7Y6S486	mangroves in China	31
Pseudomonas sp. MWU12-2323	A0A6I1NHB1	wild cranberry bog in the USA	102
Rhizobium fredii	G9A374	primary deep peat from Malaysia	43

Rhodobacterales bacterium	A0A5D0VUF8	peat forest in Malaysia	43
Rhodoplanes roseus	A0A327KD58	primary deep peat in Malaysia	43,82
Rhodovulum kholense	A0A2T5UAD1	mangrove forest in India	103
Saccharophagus degradans DSM 17024	Q21NZ9	salt marsh in the USA	82,104
Sinorhizobium medicae	A6UM82	primary deep peat in Malaysia	43
Sinorhizobium meliloti	A0A0E0UJB2	primary deep peat in Malaysia	43
Sorangium cellulosum So0157-2	S4Y1J2<br>S4XVF0	alkaline lake in China	82,105
Stenotrophomonas maltophilia	A0A0D0IVP7	mangrove soil in China	37
Stenotrophomonas rhizophila	A0A023Y3L4	mangroves in China	31,106

Table S2. Bacterial species indigenous to wetlands which harbor a TYR gene within their respective genome. The column “sampling site” indicates the location from which the organisms have been identified, as it is reported in the respective reference (“Ref.” column). The TYR protein sequences deposited in the UniProt databank[107] can be accessed *via* the identifiers reported in the “UniProt ID” column. Some organisms carry more than one TYR gene within their genome, which is indicated by multiple UniProt IDs. The organisms are grouped according to their phylum and are listed in alphabetical order.

1st (HisA1)	2nd (HisA2) and 3rd (HisA3)	4th (HisB1) and 5th (HisB2)	6th (HisB3)
B2ZB02 IAWHGAA 45	NAAHMSS------AFLPWHRE 71	-QLHNRVHRWV 211	FLHHAN 235
Q2T7K1 GGYHGEP 59	YCNHGNV------LFPTWHRM 95	ESPHNDIHLAV 283	FFHHCN 323
Q83WS2 VRTHNEF 41	RTGHRSP------SFLPWHRR 65	VNLHNRVHVWV 197	WLHHAY 220
A0A3N5X831 ANMHAHF 117	RCEHGTE------PFLTWHRM 163	LTPHGAVHCAL 266	YTHHTN 294
A0A2U3KIR3 GAIHGEY 52	QCQHQTW------YFPPWHRG 106	NNPHNLVHVYV 249	YLHHAN 280
A0A2U3LIS9 ARVHA-- 242	QCQHGWE------EFLTWHRV 258	-NIHNLIHNFS 442	WGHHSN 485
A0A1I6M5T1 ALIHQ-- 214	HCQHGWE------RFLPWHRV 230	QNPHNTMHIWT 406	WCIHVN 467
A0A239DL37 AAIHNEF 52	QCQHGSW------FFLPWHRG 103	NNPHGHVHLDV 241	YVHHAN 268
A0A3N5LM39 TRRHMLA 39	NVAHNGP------SFFPWHRS 68	ERPHNLVHTTI 195	WLHHAN 216
A0A6I3M9Z3 AALHGTP 159	NCQHGTW------YFLPWHRI 183	RSPHNAVHNAV 313	WLHHAN 335
A0A6L7GYJ8 AALHGRN 58	ACQHGSW------FFLAWHRM 86	DAPHGSVHVLV 208	WLHHAN 243
A0A1G4V5R8 TAIHWDD 59	KCQHGTW------FFLPWHRM 84	AAPHGSVHNSV 211	WLHHAN 233
A0A370D666 AAIHGRG 59	NCQHGSW------FFMPWHRM 88	DVPHGNVHVLV 210	WCHHAN 244
A0A4V2PI10 REMHRER 157	RQAHGFS------GFLAWHRA 175	GDPHGAAHSSF 302	FLLHSN 324
A0A7W9DMW4 VQLHVDV 42	RVGHINP------GFLPWHRQ 69	GKLHGAGHQWV 229	FLHHCF 250
A0A0B5F6T3 VRTHQEF 39	RVAHRGP------SFLPWHRR 63	VNLHNRVHQWV 195	WLHHCF 216
A0A0B5EPG7 VARHIAQ 42	RLAHMTP------TFFPWHRR 66	LRNHNRVHRWV 206	WLHHSF 227
A0A5J4L7U6 ISTHNAF 41	RTGHRSP------SFLPWHRR 65	PNLHNRVHVWV 197	WLHHCF 218
A0A1S9P1Z7 VTTHNAF 41	RTGHRSP------SFLPWHRR 65	VNLHNRVHVWV 197	WLHHAY 218
A0A1S9NU67 VRMHINY 42	RTAHMAP------SFLPWHRR 66	WRTHNRVHRWV 207	WLHHAY 228
S3ZAM0 VSTHNDF 41	RVGHRSP------SFLPWHRR 65	VNLHNRVHVWV 197	WLHHAY 218
S3ZGJ1 VRTHIDF 41	RVAHMTP------SFLPWHRR 65	WRTHNRVHRWV 205	WLHHAF 226
A0A328J9V5 VVMHREY 41	RPAHMTS------SFFPWHRQ 65	VGLHNRVHQWV 206	WLHHSF 227
A0A0M8QIS7 VRTHIAY 42	RTAHMAP------SFLPWHRR 66	WRNHNRVHRWI 207	WLHHAF 228
A0A7W9H3D1 VRTHIEY 42	RAAHMAP------SFLPWHRR 66	WHTHNRVHRWV 207	WMHHAF 228
A0A7W9HDB9 VTTHNAF 41	RTGHRSP------SFLPWHRR 65	VNLHNRVHVWV 197	WLHHAY 218
A0A7X0HLM5 VSLHGRF 41	RAAHMTP------SFFPWHRQ 65	GTNHNRVHRWV 205	WLHHAF 226
A0A7X0HJ80 VTTHNAF 41	RVGHRSP------SFLPWHRR 65	VNLHNRVHTWV 197	WLHHAF 218
A0A0N0H3U4 ARMHGVE 50	QCPHGGW------YFLPWHRA 79	LTPHGLIHDGV 246	WLHHAN 268
B5GYQ0 VAAHRLR 41	YVGHFGP------SFLPWHRK 65	MSMHNRVHTWV 198	WLHHAF 219
B5H1U8 VRTHGEF 41	RPAHMTP------SFFPWHRL 65	WRNHNRVHRWV 204	WLHHAF 225
A0A6N9UN96 VRTHIEY 42	RTAHMAP------SFLPWHRR 66	WRNHNRVHRWV 207	WLHHAF 228
A0A1S2Q1S7 VIWHIRA 59	NYAHRGP------VFLPWHRL 88	PWTHNQVHLWT 253	FLHHCN 274
A0A124HPH4 VRLHIDF 42	RTAHMAP------SFLPWHRR 66	WKNHNRVHRWV 207	WLHHAF 228
A0A7K3Q557 VRTHIEY 42	RTAHMAP------SFLPWHRR 66	WHNHNRVHRWV 207	WLHHAF 228

A0A7M2SPF7	VRLHIAH 42	RAAHMTP------SFLPWHRM 66	WRNHNRVHRWV 205	WLVHAF 226
A0A7M2SBB5	VTTHNAF 41	RTGHRSP------SFLPWHRR 65	VNLHNRVHVWV 197	WLHHAY 218
A0A4Y3S0B0	VRTHIDH 42	RTAHMAP------SFLPWHRR 66	FRIHNRVHRWV 207	WLHHSF 228
A0A4Y3RCW8	VTTHNAF 42	RVGHRSP------SFLPWHRR 65	VNLHNRVHVWV 197	WLHHAF 218
A0A0U3KNS5	VVMHREY 41	RPAHMTS------SFFPWHRQ 65	VGLHNRVHQWV 206	WLHHSF 227
A0A101SXR6	VRMHIDH 42	RTAHMAP------SFLAWHRR 66	WRNHNRVHRWV 207	WLHHAF 228
A0A101SWH6	VTTHNAF 42	RTGHRSP------SFLPWHRR 65	VNLHNRVHVWV 197	WLHHAY 218
A0A2X2M3Y0	VVMHREY 41	RPAHMTS------SFFPWHRQ 65	VGLHNRVHQWV 206	WLHHSF 227
A0A2X2M5T2	VLAHRTR 41	YVGHFGP------SFLPWHRK 65	MSMHNRVHTWV 198	WLHHAF 219
A0A640TR07	ISTHNAF 41	RTGHRSP------SFLPWHRR 65	PNLHNRVHVWV 197	WLHHCF 218
A0A285D923	VTLHRQY 41	RPAHMTP------SFFPWHRQ 65	IANHNKVHRWI 205	WLHHAF 226
A0A0D4DDN2	ITTHNAF 41	RTGHRSP------SFLPWHRR 65	PNLHNRVHVWV 197	WLHHCF 218
A0A1J4PWI9	VRTHIAY 42	RAAHMAP------SFLPWHRR 66	WHNHNRVHRWV 207	WLHHAY 228
A0A1J4NW81	VTTHNAF 41	RTGHRSP------SFLPWHRR 65	VNLHNRVHVWV 197	WLHHAF 218
A0A1J4P798	VRTHLEY 42	RTAHMAP------SFLPWHRK 66	WHTHNRVHRWV 207	WLHHAF 228
A0A1S2PQK3	VHIHITH 42	RTAHMTP------SFLPWHRK 66	WHNHNRVHHWV 207	WLHHAF 228
A0A1S2PKP4	VRTHNEF 42	RTGHRSP------SFLPWHRR 65	VNLHNRVHVWV 197	WLHHAY 218
A0A2S1IMI8	VTTHNAF 42	RTGHRSP------SFLPWHRR 65	VNLHNRVHVWV 197	WLHHAY 218
A0A2S1ICI5	VRLHIAH 42	RAAHMAP------SFLPWHRR 66	WRNHNRVHRWV 205	WLVHAF 226
A0A1D8SP36	VRTHIEY 42	RTAHMAP------SFLPWHRR 66	WRNHNRVHRWV 207	WLHHAF 228
A0A7W7LLS9	VRTHNEF 41	RVGHRSP------SFLPWHRR 65	VNLHNRVHVWI 197	WLHHCF 218
A0A1S6JCQ5	VRMHVDH 42	RTAHLAP------SFLPWHRR 66	WRNHNRVHRWV 207	WLHHAF 228
A0A345I2D0	VRLHDEY 41	RAGHMAP------SFLPWHRR 65	GRNHNRVHRWV 204	WLHHSF 225
A0A191UXZ3	VRTHIEY 42	RTAHMAP------SFLPWHRR 66	WRNHNRVHRWV 207	WLHHAF 228
A0A5M8GTV4	VVMHREY 42	RPAHMTS------SFFPWHRQ 65	VGLHNRVHQWV 206	WLHHSF 227
A0A221NXB2	VRMHIDY 42	RAAHMTP------SFLPWHRR 66	WHNHNRVHRWV 207	WLHHAF 228
A0A221P6L7	VRTHNEF 41	RTGHRSP------SFLPWHRK 65	VNLHNRVHVWV 197	WLHHAY 218
A0A5B8IB42	VAAHRLR 41	YVGHFGP------SFLPWHRK 65	MSMHNRVHTWI 198	WLHHAF 219
A0A5B8JHY3	VRMHGEF 41	RAAHMTP------SFFPWHRR 65	WRTHNRVHRWV 205	WLHHAF 226
A0A510D900	VRTHVAY 42	RTAHMAP------SFLPWHRR 66	WHNHNRVHRWV 207	WLHHAY 228
A0A6I5MM61	VRTHIEY 42	RTAHMAP------SFLPWHRR 66	WRNHNRVHRWV 207	WLHHAF 228
A0A0J6XJT5	VRTHDKY 41	RVGHMSP------SFFPWHRR 65	WRNHNKVHQWI 206	WLHHAF 227
A0A0J7AI25	VTTHNGF 41	RVGHRSP------SFLPWHRR 65	ANLHNRVHVWV 197	WMHHAY 218
A0A2P2GLL6	VTTHNAF 41	RVGHRSP------SFLPWHRR 65	VNLHNRVHVWV 197	WMHHAF 218
A0A2P2GJB2	VRTHIDH 42	REAHMCP------SFLPWHRR 66	FRLHNRVHRWV 207	WLHHAY 228
A0A1S2PK94	VHIHIVH 42	RSAHMTP------SFLPWHRK 66	WHNHNRVHHWV 207	WLHHAF 228
A0A1S2P7Y5	VRTHNEF 41	RTGHRSP------SFLPWHRR 65	VNLHNRVHVWV 197	WLHHAY 218
A0A024YX16	VRTHDTY 41	RVGHMSP------SFFPWHRL 65	WRNHNKVHQWI 206	WLHHAF 227
A0A024YX26	VTTHNGF 41	RVGHRSP------SFLPWHRR 65	VNLHNRVHVWV 197	WMHHAF 218
A0A4V3S5K9	VRTHVAY 42	RTAHMAP------SFLPWHRR 66	WHNHNRVHRWV 207	WLHHAY 228
A0A2S3Y8X7	VTTHNAF 41	RVGHRSP------SFLPWHRR 65	VNLHNRVHVWV 197	WLHHAF 218
A0A2S3XTZ2	VVMHREY 41	RPAHMTP------SFFPWHRR 65	VANHNRVHQWV 206	WLHHSF 227
A0A516RAC2	VRTHIDF 42	RVAHMTP------TFLPWHRR 66	WRNHNRVHRWV 207	WLNHAF 228
A0A516R853	VSTHNDF 41	RVGHRSP------SFLPWHRR 65	VNLHNRVHVWV 197	WLHHAY 218
Q9F1K7	VTTHNAF 41	RVGHRSP------SFLPWHRR 65	VNLHNRVHVWV 197	WMHHAF 218
F2RIZ9	VTTHNAF 41	RVGHRSP------SFLPWHRR 65	VNLHNRVHVWV 197	WLHHAF 218
F2RLH9	VRTHIDH 42	RVAHMTP------SFLPWHRK 66	FRIHNRVHRWV 207	WLHHSF 228
A0A1V0UKR2	VVMHREY 41	RPAHMTP------SFFPWHRQ 65	VGLHNRVHQWV 206	WLHHSF 227
A0A1V0UF35	VSTHRTR 41	YVGHFGP------SFLPWHRK 65	MSMHNRVHTWV 198	WLHHAF 219
A0A0L8M4B5	VTTHNGF 42	RVGHRSP------SFLPWHRR 65	ANLHNRVHVWV 197	WMHHAF 218
A0A0L8M4C5	VRTHDKY 41	RVGHMSP------SFFPWHRR 65	WRNHNKVHQWI 206	WLHHAF 227
D9XAS3	VRMHIEY 42	RAAHMAP------SFLPWHRR 66	WRNHNRVHGWV 207	WLHHAF 228
D9X674	VTTHNAF 41	RTGHRSP------SFLPWHRR 65	VNLHNRVHVWV 197	WLHHAY 218
A0A1H0A5D2	ITTHNAF 41	RTGHRSP------SFLPWHRR 65	PNLHNRVHVWV 197	WLHHCF 218
A0A1H0C516	VTTHNAF 41	RVGHRSP------SFLPWHRR 65	VNLHNRVHVWV 197	WLHHAF 218
A0A1N6FBL6	AAIHGEN 52	QCQHQSW------YFAPWHRG 102	TNPHNFVHNDV 231	YLHHCN 260
A0A383RI28	AGLHNDP 49	PCEHGND------LFFAWHRA 67	IGPHNYMHPIY 168	WSFHAF 191
Q6EH49	VDVHRKH 41	HPIHQSA------MFLPWHRE 58	--LHNGPHNWV 155	WLHHSN 176
D5DHA8	IAWHGAA 45	NAAHMSS------AFLPWHRE 71	PQLHNRVHRWV 211	FLHHAN 233
A0A0F6J033	VYWHLMS 52	GSAHGGP------AFLPWHRY 76	GRIHNQVHLWI 238	FLHHAN 263
A0A2C5KJN2	VYWHLQS 52	GWAHRGP------AFLPWHRY 76	GSIHNKVHVWV 236	FLHHAN 261
H0UHZ0	VHWHHAV 25	NGAHRGP------AFLPWHRE 56	TQLHNRVHLWV 208	FLHHCF 240
A0A1D7MFL7	AGLHNDP 50	PCEHGND------LFLAWHRA 68	LGPHNYMHSFY 169	WSFHAF 192
A0A1D7MCL4	AGYHWI- 43	FCPHGES------IFLPWHRV 61	EGPHGDIHGWV 172	WSHHAN 197
A0A6H1TJ97	IAWHGAA 45	NAAHMSS------AFLPWHRE 71	PQLHNRVHRWV 211	FLHHAN 233
D8PI13	ANVH--- 111	-QVHFSW------QFFAWHRA 133	GTPHGAVH--- 234	YAHHAN 259

A0A6M5YW59	ANFHNT- 82	SCAHRNW------FFFPWHRA 97	GGPHNIIHEYV 206	WVHHAN 236
L0D705	VHWHHHV 41	NGAHLGP------AFLPWHRE 72	SQLHNRVHLWV 225	FLHHCF 246
A0A6A1RQV4	GAMHGIT 65	QCQHATW------FFLPWHRG 125	QYPHNIMHVLV 273	YVHHAN 300
A0A1X7NGR1	ALVH--- 81	HCPHGAE------DFVHWHRY 98	GTPHNTGHVVV 217	WLHHCM 241
G7Z6L5	AYIHSLP 73	TCPHGSP------YFLPWHRW 134	TQPHDNVHDGV 262	YMHHCQ 284
A0A6N1APE8	AFVH--- 75	DCPHGNW------WFYVWHRG 92	GQPHNLVHNCI 282	FLHHAN 313
A0A2B8BFB1	AFVH--- 75	DCPHGNW------WFYVWHRG 92	GQPHNLVHNCI 282	FLHHAN 313
A0A5A9EZ76	AFVH--- 75	DCPHGNW------WFYVWHRG 92	GQPHNLVHNCI 282	FLHHAN 313
A0A1L3FP25	ANIHWTP 81	KCPHHMY------DFLPWHRV 154	SAPHDQVHGRI 272	YLHHSN 301
A0A1L3FF99	ALIHQ-- 207	HCQHGWE------RFLPWHRA 223	QNPHNTMHIWT 412	WPIHVN 496
A0A1L3FQJ7	AGMHGYH 49	QCQHGSW------YFLPWHRG 91	SQPHDQVHGLV 220	WLHHAN 256
A0A346R2L6	AGYHWLP 132	YCQHHVP------GYNPWHRA 150	IAAHDGGHGAI 263	WFFHSN 285
A0A346R3B5	ALIHQ-- 218	HCQHGWE------RFLPWHRV 234	QNPHNTMHIWT 423	WPVHAN 483
A0A346QXY8	VFWHLLA 70	NAAHGGP------IFLPWHRM 93	PQLHNRVHVWV 237	FLNHCN 258
A0A2A7SC70	AFIH--- 84	DCPHGNW------WFYVWHRG 101	GLPHNKVHNYI 294	FLHHAN 325
A0A6J5CVK2	GGFHGEP 59	YCNHGNV------LFPTWHRA 88	EEPHNNIHLAV 279	YFHHCF 317
A0A6G8NL20	VNVH--- 78	NCPHMVP------YFLAWHRG 95	SAPHNPVHNII 189	YLHHAN 210
A0A1S1WVR1	WYTHFVR 85	TCQAHSS-GQREDFFLPWHRM 133	MNPHGALHVDV 241	WLHHCN 265
A0A1S1WVB3	AAVHGTN 56	QCQHATW------LFLPWHRM 86	MVPHDVIHSDI 214	WLHHAN 236
A0A1D9LLW6	WYTHFVR 84	TCQAHSS-GQREDFFLPWHRM 132	MNPHGALHVDV 237	WIHHCN 261
A0A1D9LEY9	AAVHGTL 46	QCQHATW------FFLPWHRV 76	MVPHDMIHSDI 202	WLHHAN 224
A0A538QY07	AAIHGLD 48	QCQHGSS------FFLPWHRM 75	LTVHNRIHGRV 194	WLHHAN 216
A0A7D3Y0J0	AQIH--- 79	TCPHGNW------FFLPWHRA 96	QGPHNYIHGFV 203	WLHHCN 224
A0A261GRE4	GAIHGYA 46	RCQHGTW------YFLPWHRM 89	SLPHNVVHTDI 218	WLHHAN 240
A0A261GVB1	GGYHGEP 59	WCNHGNV------LFPTWHRI 95	EQPHNDVHLAV 289	FFHHCN 328
A0A7H8RK67	AWVHNCT 93	ECEHQKN------TFLQWHRA 158	TKIHNHMHA-- 267	YAFHNF 292
A0A250IQN3	AGLHG-- 64	YCPHGNL------SFLIWHRP 83	EQPHNGLHGWV 198	WVHHCF 220
A0A1B4YH80	AAIHGIE 32	QCQHHGW------YFVPWHRG 75	QVPHDVVHGRV 205	WLHHAN 241
A0A2S6H7D7	ALYHQ-- 212	HCQHGWE------RFLPWHRA 228	QNPHNTMHIWT 416	WPIHVN 493
A0A6B8LR17	VVWHVTA 59	NAAHRGP------IFAPWHRV 87	TSMHNLVHVWI 239	FLNHCN 260
A0A7X0B1W6	AFIH--- 80	DCPHGNW------WFFIWHRG 97	GQPHNLTHNCI 289	FLHHSN 320
A0A4R7BDA0	AAIHGFD 50	QCQHGSW------YFLPWHRG 92	TQPHDWVHGLV 218	WLHHSN 249
J2Y1W2	AGYHWFP 154	YCMHHVP------GYNPWHRA 173	IAAHDSGHNSI 286	WFFHCN 308
A0A4U3F4Z1	VQIHKNS 51	PNPHRSP------LFYPWHRI 74	TELHNPVHAWI 199	FLHHCY 220
A0A7Y6S486	AYIHT-- 161	WCLHYQE------AFALWHRA 177	EQPHDNYHGWI 346	CSYHAN 368
A0A6I1NHB1	WYTHAMP 82	TCEPHASKGVNSNAFLPWHRM 132	GNPHGALHGDV 243	WLHHAN 267
G9A374	GFIH--- 80	DCPHGDW------WFTSWHRG 97	GQPHNRVHMSV 273	FLHHCN 300
A0A5D0VUF8	GAIHGCD 48	QCQHQSW------FFLPWHRG 92	QNPHNVVHGYV 217	WLHHCN 240
A0A327KD58	ALVH--- 84	DCPHGNW------WFLVWHRA 101	GQPHNRVHNCV 269	FLHHAN 299
A0A2T5UAD1	AMIH--- 77	DCPHGNW------WFFVWHRP 94	GQPHNKVHNDL 277	FMHHCN 302
Q21NZ9	GKLHG-- 218	VCAHGFE------QFLTWHRM 234	MEPHNTIHIWV 436	WPHHVN 483
A6UM82	GFIH--- 80	DCPHGDW------WFTSWHRG 97	GQPHNRVHMSV 273	FLHHCN 300
A0A0E0UJB2	GFIH--- 80	DCPHGDW------WFTSWHRG 97	GQPHNRVHMSV 273	FLHHCN 300
S4Y1J2	ANVH--- 56	ENIHGNY------WFLPWHRA 82	FGTHNRVHQWI 189	FAHHAN 221
S4XVF0	ANVH--- 56	ENIHGNY------WFLPWHRA 82	FGTHNRVHQWI 189	FAHHAN 221
A0A0D0IVP7	ADIHGGS 74	NCQHGNW------YFLPWHRA 93	SGPHNYVHDEV 226	FLHHAN 247
A0A023Y3L4	AFVH--- 83	DCPHGNW------WFYVWHRG 100	GFPHNKVHNCI 292	YLHHAN 323

Table S3. Multiple sequence alignment. Sections of a multiple sequence alignment of the TYR sequences present within the respective genome of bacteria indigenous to wetlands showing the six histidine residues involved in the coordination of the type III di-copper center. All six His residues show perfect conservation among the bacterial TYR sequences identified herein. The first three rows (highlighted in green) represent the Cu coordinating histidines of previously kinetically and structurally (*via* X-ray crystallography) characterized bacterial TYRs, which allowed to verify the conserved histidines highlighted in yellow as the Cu coordinating histidines.

Organism	k_{cat} (s^{-1})	K_m (mM)	k_{cat}/K_m (s^{-1} mM^{-1})	Ref
Aeromonas media	n.r.	*L*-DOPA: 1.16 - 0.64	n.r.	108
Bacillus aryabhattai	*L* -tyrosine: 2.55<br>*L* -DOPA: 3.78	*L* -tyrosine: 0.163<br>*L* -DOPA: 0.288	*L* -tyrosine: 15.6<br>*L* -DOPA: 13.1	4
Bacillus megaterium	*L* -tyrosine: 1.29[109]– 4.0[110]<br>*L* -DOPA: 10.1[5] - 44.1[110]	*L* -tyrosine: 0.075[5] – 0.038[109]<br>*L*-DOPA: 0.35[5] – 0.8[110]	*L*-tyrosine: 33.9<br>*L*-DOPA: 28.9 – 55.1	5,109,110
Bacillus thuringiensis	*L*-tyrosine: 6.7<br>*L*-DOPA: 28.3	*L*-tyrosine: 0.56<br>*L*-DOPA: 0.77<br>Dopamine: 0.89	*L*-tyrosine: 12.0<br>*L*-DOPA: 36.8	6
Burkholderia thailandensis	*L*-tyrosine: 398<br>*L*-DOPA: 487	*L*-tyrosine: 0.59<br>*L*-DOPA: 0.83	*L*-tyrosine: 675<br>*L*-DOPA: 587	7
Laceyella sacchari	*L*-tyrosine: 3.1<br>*L*-DOPA: 1177<br>dopamine: 46	*L*-tyrosine: 0.055<br>*L*-DOPA: 4.5<br>dopamine: 1.5	*L*-tyrosine: 56.4<br>*L*-DOPA: 262<br>dopamine: 30.7	111
Marinomonas mediterranea	n.r.	n.r.	n.r.	
Pseudomonas aeruginosa	*L*-tyrosine: 1.38<br>*L*-Dopa: 0.13	*L*-tyrosine: 1.30<br>*L*-Dopa: 0.75	*L*-tyrosine: 1.06<br>*L*-DOPA: 0.173	112
Pseudomonas putida F6	n.r.	*L*-tyrosine: 0.23<br>*L*-Dopa: 0.33	n.r.	8
Ralstonia solanacearum	*L*-tyrosine: 1.42	*L*-tyrosine: 2.1	*L*-tyrosine: 0.676	113
Rhizobium etli	n.r.	*L*-DOPA: 2.44<br>*L*-tyrosine: 0.19	n.r.	10
Streptomyces albus	*L*-tyrosine: 9.0<br>*L*-DOPA: 1.26	*L*-tyrosine: 0.5<br>*L*-DOPA: 7.8	*L*-tyrosine: 18.0<br>*L*-DOPA: 0.162	11
Streptomyces antibioticus	*L*-DOPA: 1300	*L*-DOPA: 8.9	*L*-DOPA: 146	13
Streptomyces avermitilis	*L*-tyrosine: 0.54<br>*L*-Dopa: 4.97	*L*-tyrosine: 0.59<br>*L*-Dopa: 2.79	*L*-tyrosine: 0.763<br>*L*-Dopa: 1.67	114
Streptomyces castaneoglobisporus	*L*-DOPA: 880	*L*-DOPA: 8.1	*L*-DOPA: 109	115
Streptomyces cyaneofuscatus	n.r.	*L*-DOPA: 1.46	n.r.	14
Streptomyces glaucescens	*L*-tyrosine: 13.2<br>*L*-DOPA: 1445	*L*-tyrosine: 0.41<br>*L*-DOPA: 5.77	*L*-tyrosine: 32.2<br>*L*-DOPA: 250	15
Streptomyces kathirae SC-1	*L*-DOPA: 171	*L*-DOPA: 0.42	*L*-DOPA: 407	16
Streptomyces michiganensis	n.r.	n.r.	n.r.	
Streptomyces REN-21	*L*-tyrosine: 81.4<br>*L*-DOPA: 1120	*L*-tyrosine: 1.25<br>*L*-DOPA: 4.14	*L*-tyrosine: 65.1<br>*L*-DOPA: 271	18
Streptomyces sp. ZL-24	*L*-tyrosine: 4.8<br>tyramine: 6.3<br>*L*-DOPA: 520<br>dopamine: 320	*L*-tyrosine: 0.60<br>tyramine: 5.6<br>dopamine: 7.4<br>*L*-DOPA: 15	*L*-tyrosine: 8.00<br>tyramine: 1.12<br>dopamine: 70.3<br>*L*-DOPA: 21.2	19
Thermomicrobium roseum	n.r.	*L*-DOPA: 0.18	n.r.	20
Verrucomicrobium spinosum	n.r.	n.r.	n.r.	

Table S4. Kinetic parameters of previously characterized bacterial TYRs. “n.r.” (not reported) indicates that the respective parameter has not been reported. Chemical structures of *L*-tyrosine, tyramine, dopamine, and *L*-DOPA are illustrated in Figure S3.

Organism	Coordinates of the sampling site	Ref.
**northern regions**		
Granulicella pectinivorans	56°51'N 82°50'E	27
Granulicella rosea	58°14'N 38°12'E	27
Azospirillum palustre	Tver Oblast, Russia.	85
Methylocystis rosea	78°56'N 11°53'E	96
Singulisphaera acidiphila	58°14'N 38°12'E	80
**tropic regions**		
Streptomyces mangrovisoli	3°48'N 103°20'E	50,116
Streptomyces malaysiense	3°48'N 103°26'E	49
Streptomyces sp. MUSC 14	3°48'N 103°20'E	58
Streptomyces ferrugineus	13°30'N 101°0'E	45
Streptomyces monashensis	1°41'N 110°11'E	51
Streptomyces pluripotens (=MUSC 137T)	3°48'N 103°26'E	55
Burkholderia paludism	3°01'N 103°39'E	88
Rhodovulum kholense	20°43'N 86°49'E	103

Table S5. Coordinates of the sampling sites of *tyr*$^{+}$ bacteria listed in Table 3 of the main manuscript. No precise coordinates are reported for *Azospirillum palustre*, thus the region of Tver Oblast (Russia) is listed, as reported by Tikhonova *et al.*[85].

3. Supplementary Figures

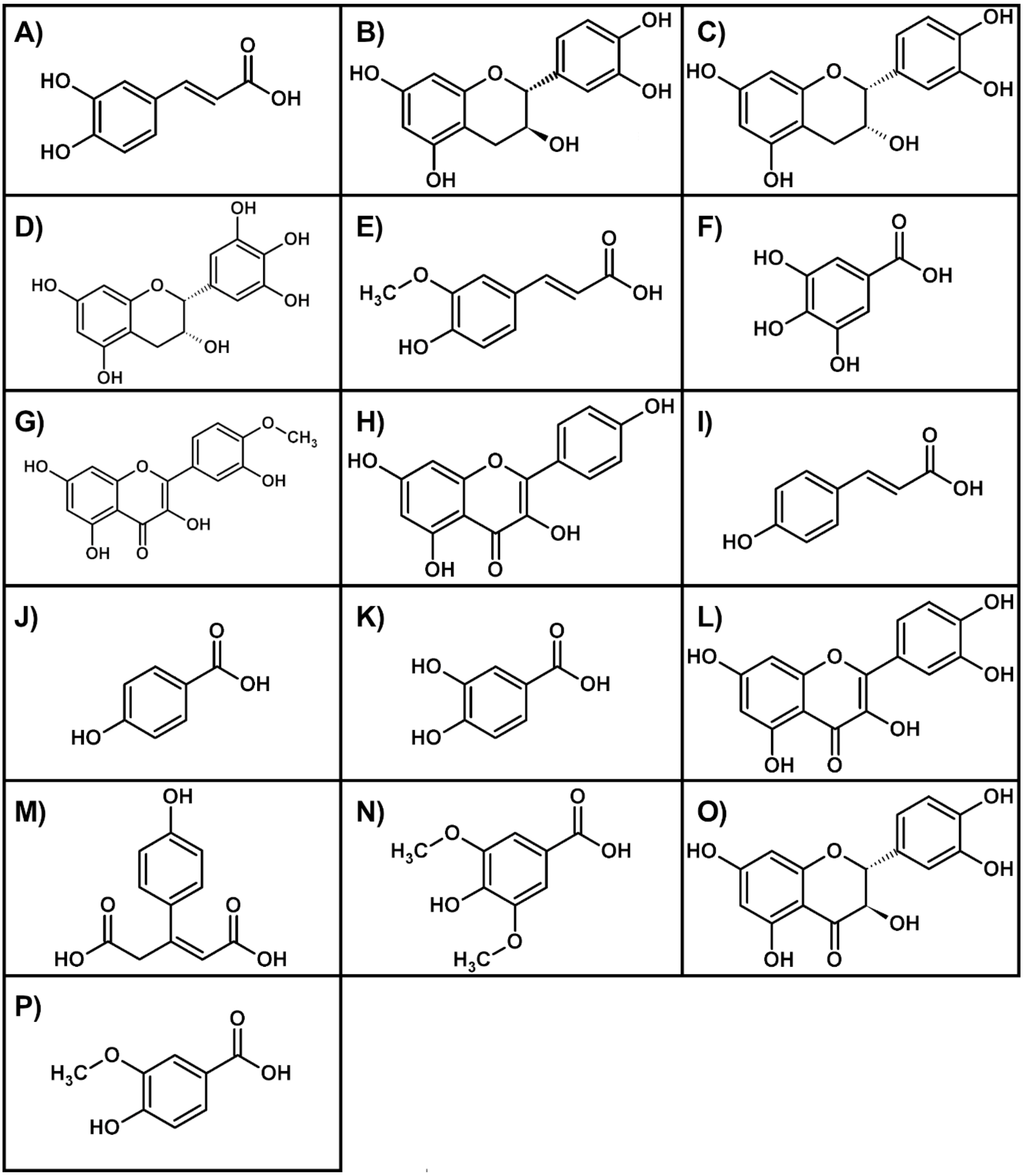

Figure S1. Phenolic substrates previously identified within wetlands. **A** = caffeic acid, **B** = (+)-catechin, **C** = (-)-epicatechin, **D** = (-)-epigallocatechin, **E** = ferulic acid, **F** = gallic acid, **G** = isorhamnetin, **H** = kaempferol, **I** = *p*-coumaric acid, **J** = *p*-hydroxybenzoic acid, **K** = protocatechuic acid, **L** = quercetin, **M** = sphagnum acid, **N** = syringic acid, **O** = taxifolin, **P** = vanillic acid. Substrates are listed alphabetically. The Figure was edited using GIMP 2.10.18 (https://www.gimp.org).

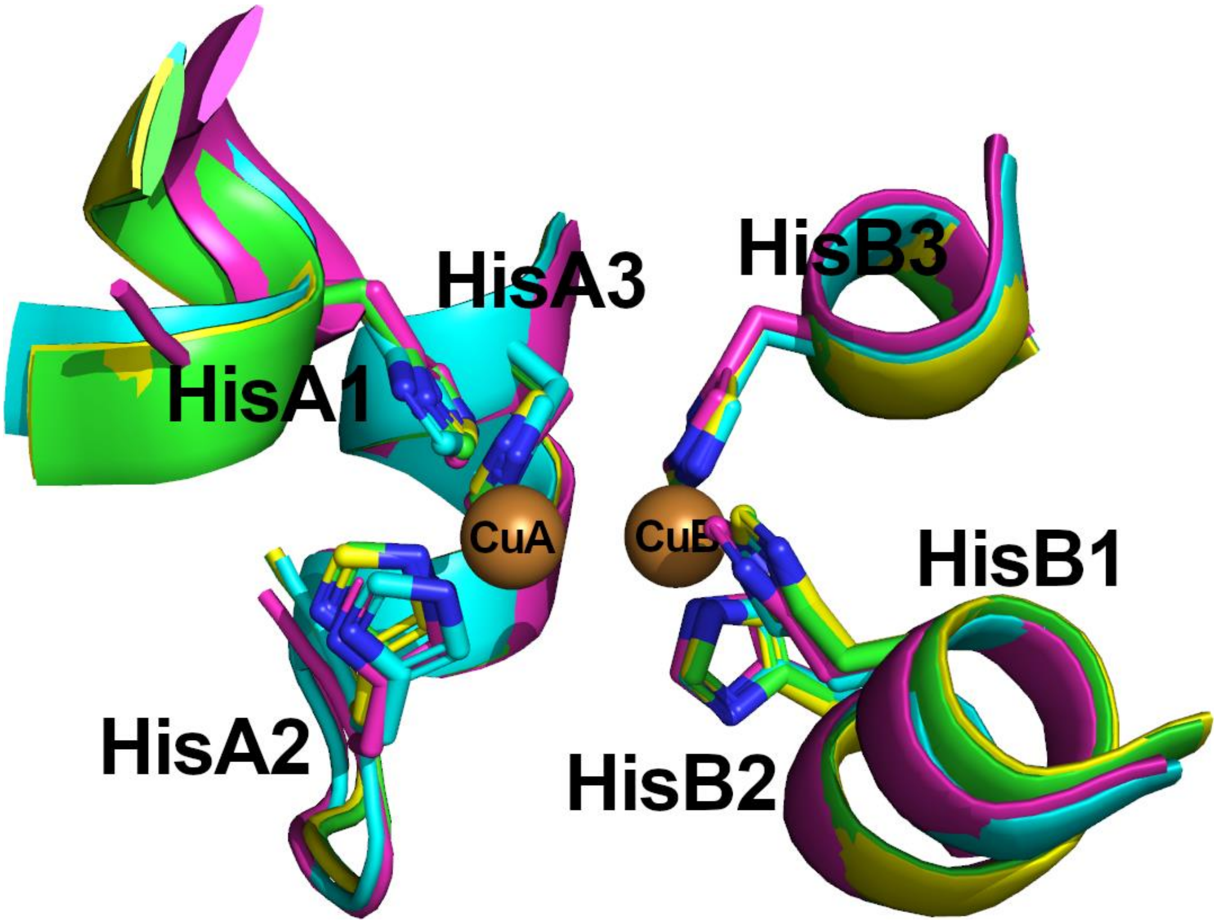

Figure S2. Coplot of the active centers of crystallographically characterized bacterial TYRs. Cyan: *Bm*TYR (*Bacillus megaterium*[117], PDB entry: 3NM8), magenta: *Bt*TYR (*Burkholderia thailandensis*[7], PDB entry: 5ZRE), yellow: *Sa*TYR (*Streptomyces avermitilis*, PDB entry: 6J2U), green: *Sc*TYR (*Streptomyces castaneoglobisporus*[118], PDB entry: 2ZMZ). The six Cu-coordinating histidine residues are highlighted as sticks. The two Cu ions are shown as brown spheres and labeled CuA and CuB. HisA1, HisA2, and HisA3 are involved in the coordination of CuA (numbered in the order in which they appear in the sequences of the respective enzymes). HisB1, HisB2, and HisB3 are involved in the coordination of CuB (numbered in the order in which they appear in the sequences of the respective enzymes). HisB1 and HisB2 are also involved in the formation of the conserved His-X-X-X-His (X = any amino acid) motif. For simplicity, only the Cu ions from the crystal structure of *Bm*TYR (PDB entry: 3NM8) are shown. In contrast to plant and fungal TYRs, the thioether bridge connecting the second copper-coordinating histidines with the sulfur of a neighboring cysteine residue is absent in bacterial TYRs[119]. The six Cu-coordinating histidine residues are present in all TYR sequences identified within the context of this review (Table S3). The Figure was created using PyMOL 2.3 and edited using GIMP 2.10.18 (https://www.gimp.org).

1) 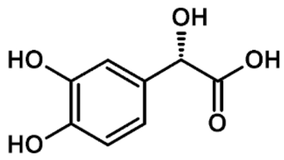	2) 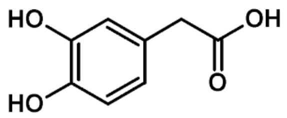	3) 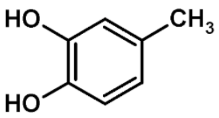
4) 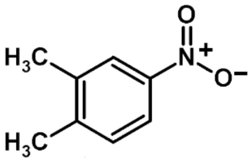	5) 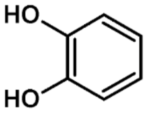	6) 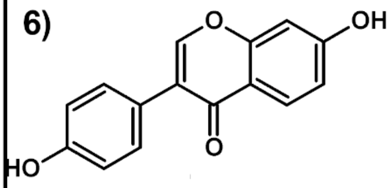
7) 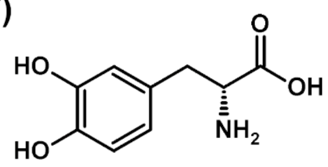	8) 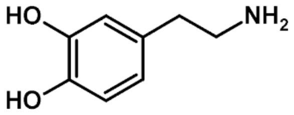	9) 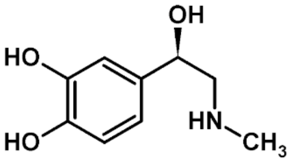
10) 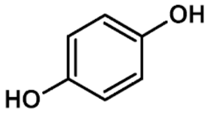	11) 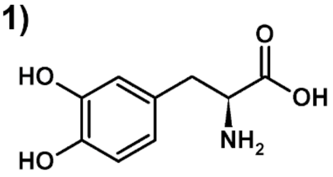	12) 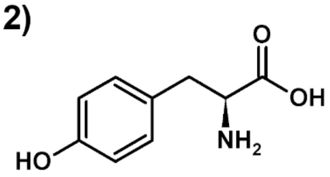
13) 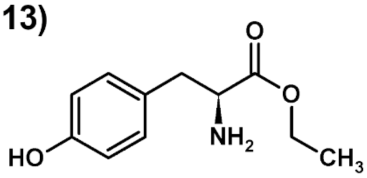	14) 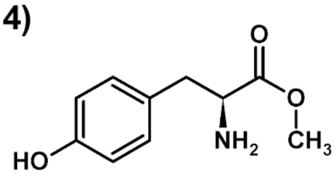	15) 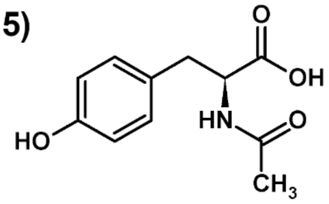
16) 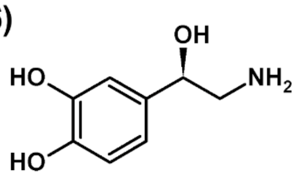	17) 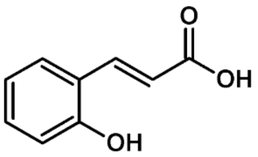	18) 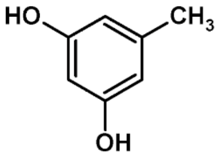
19) 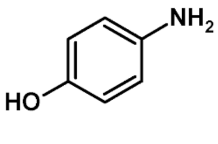	20) 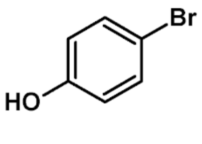	21) 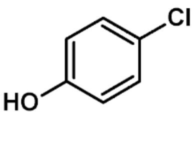

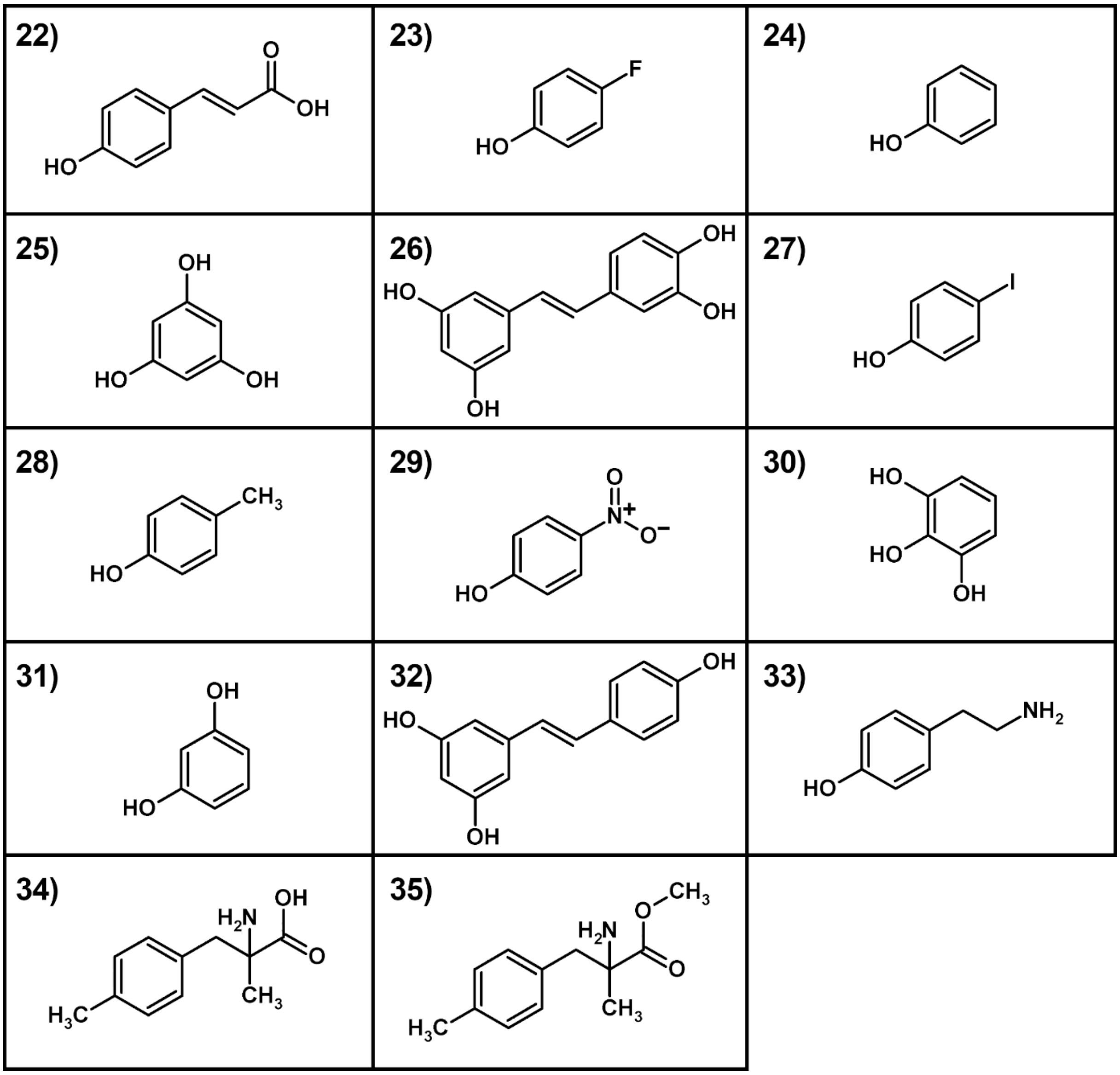

Figure S3. Phenolic compounds accepted as substrates by bacterial TYRs. **1** = 3,4-dihydroxymandelic acid, **2** = 3,4-dihydroxyphenylacetic acid, **3** = 4-methyl catechol, **4** = 4-nitrocatechol, **5** = catechol, **6** = daidzein, **7** = *D*-DOPA, **8** = dopamine, **9** = epinephrine, **10** = hydroquinone, **11** = *L*-DOPA, **12** = *L*-tyrosine, **13** = *L*-tyrosine ethyl ester, **14** = *L*-tyrosine methyl ester, **15** = N-acetyl-*L*-tyrosine, **16** = norepinephrine, **17** = *o*-coumaric acid, **18** = orcin, **19** = *p*-aminophenol, **20** = *p*-bromophenol, **21** = *p*-chlorophenol, **22** = *p*-coumaric acid, **23** = *p*-fluorophenol, **24** = phenol, **25** = phloroglucinol, **26** = piceatannol, **27** = *p*-iodophenol, **28** = *p*-methylphenol, **29** = *p*-nitrophenol, **30** = pyrogallol, **31** = resorcinol, **32** = resveratrol, **33** = tyramine, **34** = α-methyl-*DL*-tyrosine, **35** = α-methyl-*DL*-tyrosine ethyl ester. Substrates are listed alphabetically. The Figure was edited using GIMP 2.10.18 (https://www.gimp.org).

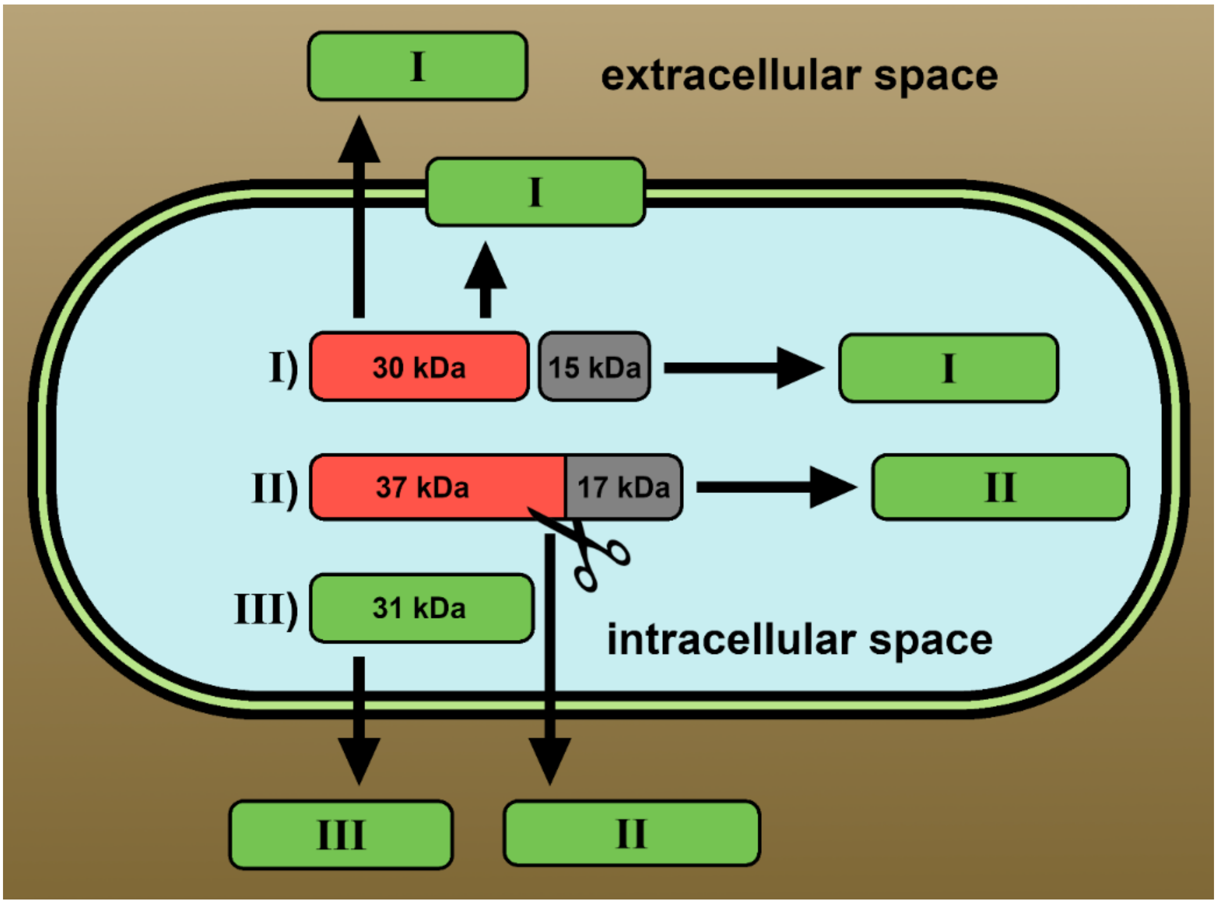

Figure S4. General architecture and cellular localization of bacterial TYRs. The Figure illustrates a bacterial cell. Red squares indicate the latent TYRs while green squares indicate the active TYRs. I) illustrates the general architecture encountered in *Streptomyces* species. Following ribosomal translation and folding, the latent TYR (red) is present in complex with a caddie protein (grey). After dissociation of the caddie protein, the active enzyme has been reported extracellularly[14,108–110], intracellularly[60,111–113], and membrane associated[114,115]. II) illustrates the general architecture present in *V. spinosum* and *R. solanacearum.* After ribosomal translation and folding, the C-terminal domain (grey) is responsible for the latency of the enzyme. Proteolytic cleavage of the C-terminal domain results in active TYR (green), which is located intracellularly (*R. solanacearum*)[103] or extracellularly (*V. spinosum*)[93]. III) illustrates the general architecture present in *Bacillus* species. After ribosomal translation and folding, the active TYR (green) is extracellularly secreted[64,107]. The Figure has been edited using GIMP 2.10.18 (https://www.gimp.org).

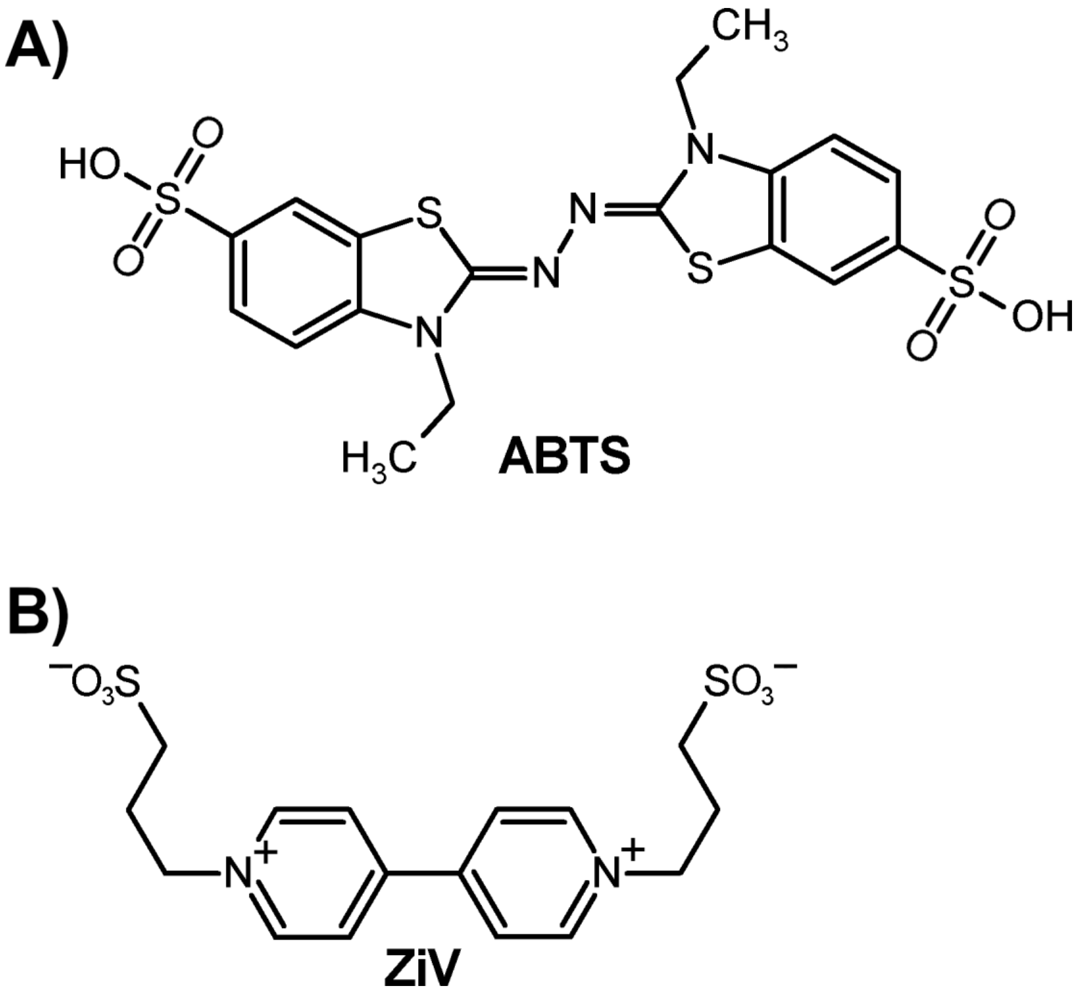

Figure S5. Chemicals used for the chronoamperometric measurement of TYR activity. Chemical structures of ABTS (2,2'-Azino-bis(3-ethylbenzothiazoline-6-sulfonic acid)) (A), and ZiV (zwitterionic viologen, N,N′-bis(3- sulfonatopropyl)-4,4′-bipyridinium) (B).

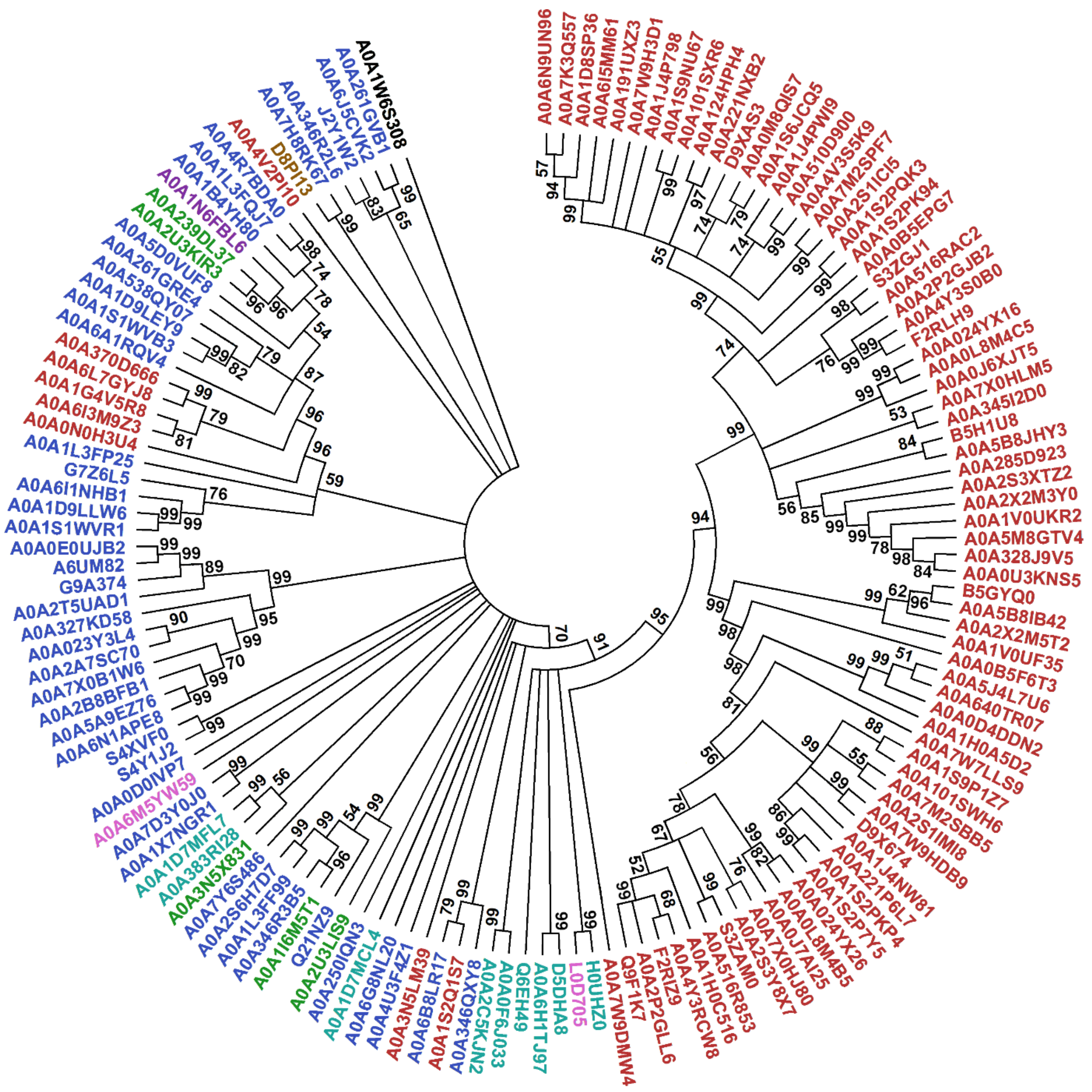

Figure S6. Phylogenetic tree of the TYRs identified within the genomes of bacterial host organisms indigenous to wetland ecosystems. The tree was rooted to the laccase from *Bacillus subtilis* (A0A1W6S308), which was used as an outgroup. TYR-identifiers (UniProt) are color-coded according to their phylum to help visualize their phylogenetic classification: green = Acidobacteria, red = Actinobacteria, purple = Bacteroidetes, cyan = Firmicutes, brown = Nitrospirae, pink = Planctomycetes, black = root (laccase from *Bacillus subtilis)*. The reliability of the internal branches was assessed by bootstrapping (400 replicates) and probability values

are reported in %. Branches corresponding to partitions reproduced in < 50 % of bootstrap replicates are collapsed. Detailed information on the computational setup is provided in the Materials and Methods section of the Supporting Information. The Figure has been edited using GIMP 2.10.18 (https://www.gimp.org).

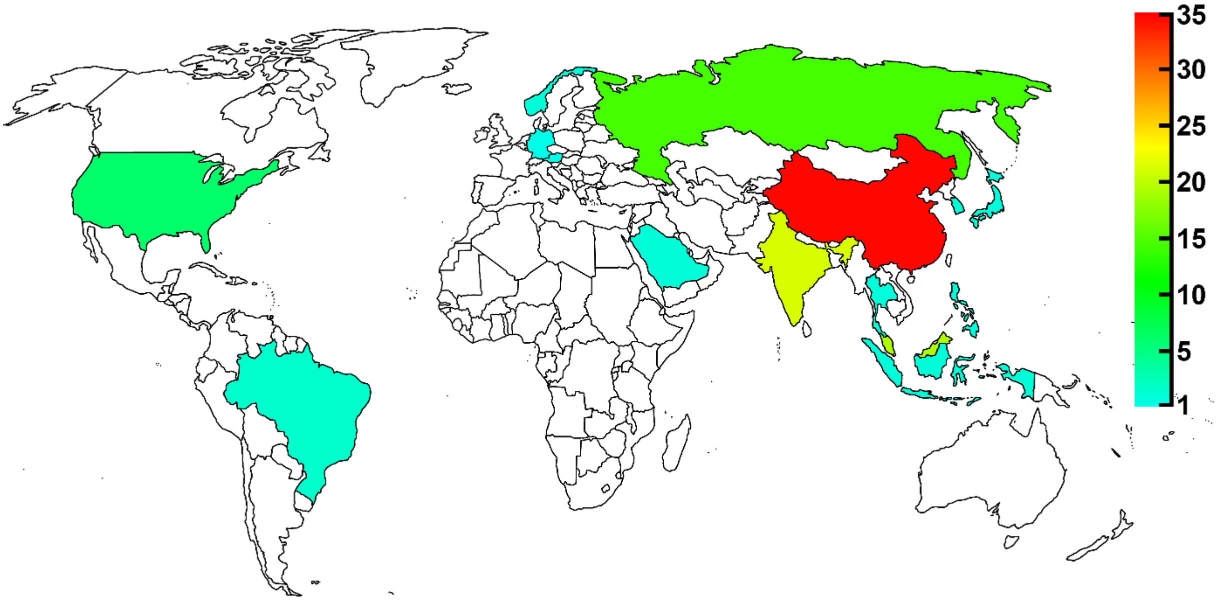

Figure S7. Global distribution of the identified *tyr*⁺ organisms. The number of *tyr*⁺ organisms identified per country is indicated by the color code. *tyr*⁺ organisms were identified from a climatic and geographic continuum ranging from high arctic regions to tropic regions, located in Asia, Europe, North America, and South America. For detailed information on the sampling sites see Table S2 in the Supplementary Information. The Figure has been edited using GIMP 2.10.18 (https://www.gimp.org).

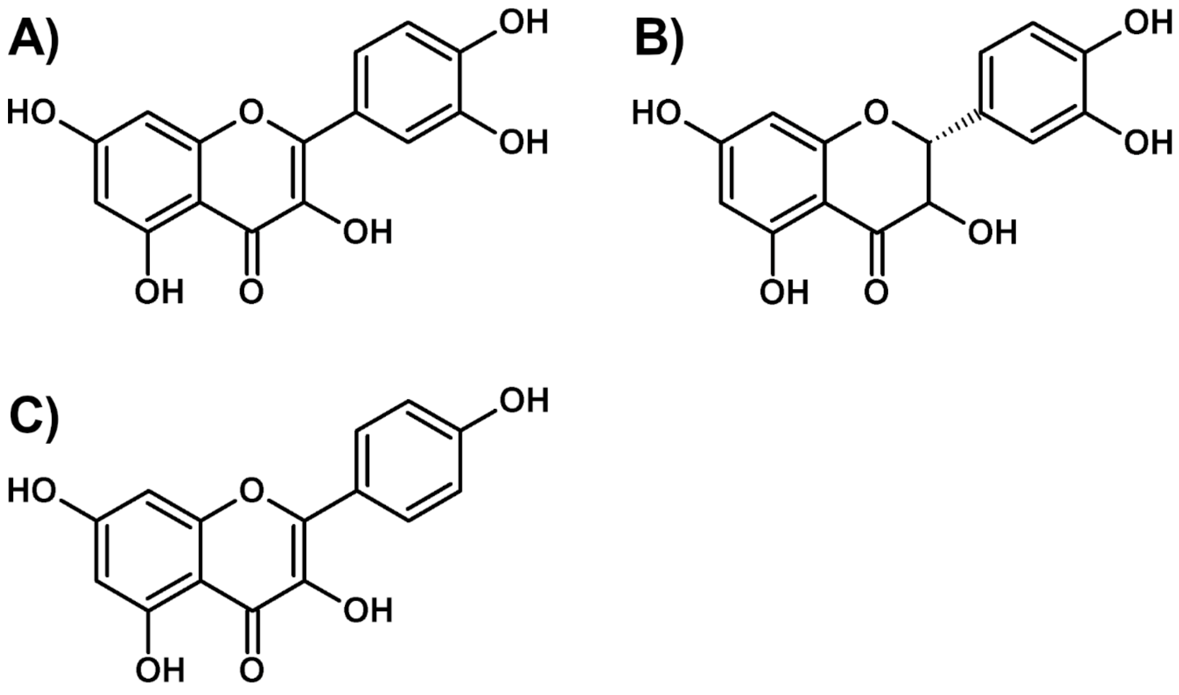

Figure S8. Chemical structures of quercetin (A), taxifolin (B), and kaempferol (C).

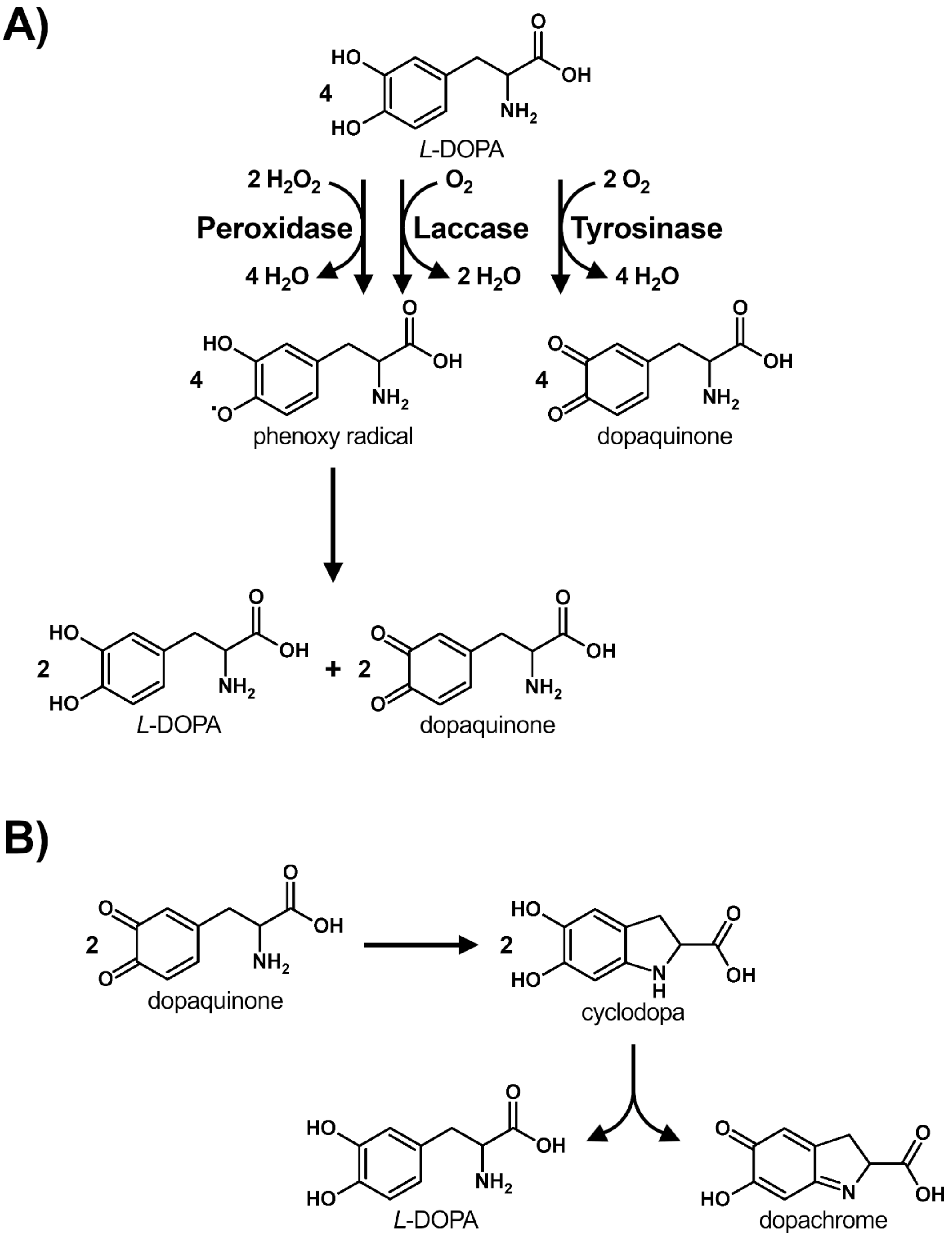

Figure S9. Oxidation of L-DOPA by laccases, peroxidases, and tyrosinases. A) Laccases[120] and peroxidases[121] generate a phenoxy radical, which, in a soil environment, can undergo a diverse scope of reactions leading (among others) to the polymerization, and depolymerization of humic substances[122,123]. In the controlled environment of an *in-vitro* assay, phenoxy radicals form dopaquinone. Tyrosinases, in contrast, directly generate dopaquinone *via* two-electron oxidation. **B)** Dopaquinone non-enzymatically forms cyclodopa, which is converted into *L*-DOPA and the chromophore dopachrome *via* redox exchange[124]. Dopachrome is detected photometrically at 480 nm[125].

4. References

(1) Kim, M.; Oh, H. S.; Park, S. C.; Chun, J. Towards a Taxonomic Coherence between Average Nucleotide Identity and 16S rRNA Gene Sequence Similarity for Species Demarcation of Prokaryotes. *Int. J. Syst. Evol. Microbiol.* **2014**, *64*, 346–351. https://doi.org/10.1099/ijs.0.059774-0.

(2) Kumar, S.; Stecher, G.; Li, M.; Knyaz, C.; Tamura, K. MEGA X: Molecular Evolutionary Genetics Analysis across Computing Platforms. *Mol. Biol. Evol.* **2018**, *35*, 1547–1549. https://doi.org/10.1093/molbev/msy096.

(3) Wan, X.; Chai, B.; Liao, Y.; Su, Y.; Ye, T.; Shen, P.; Chen, X. Molecular and Biochemical Characterization of a Distinct Tyrosinase Involved in Melanin Production from *Aeromonas media*. *Appl. Microbiol. Biotechnol.* **2009**, *82*, 261–269. https://doi.org/10.1007/s00253-008-1742-5.

(4) Wang, F.; Xu, Z.; Wang, C.; Guo, Z.; Yuan, Z.; Kang, H.; Li, J.; Lu, F.; Liu, Y. Biochemical Characterization of a Tyrosinase from *Bacillus aryabhattai* and its Application. *Int. J. Biol. Macromol.* **2021**, *176*, 37–46. https://doi.org/10.1016/j.ijbiomac.2021.02.042.

(5) Shuster, V.; Fishman, A. Isolation, Cloning and Characterization of a Tyrosinase with Improved Activity in Organic Solvents from *Bacillus megaterium*. *J. Mol. Microbiol. Biotechnol.* **2009**, *17*, 188–200. https://doi.org/10.1159/000233506.

(6) Liu, N.; Zhang, T.; Wang, Y. J.; Huang, Y. P.; Ou, J. H.; Shen, P. A Heat Inducible Tyrosinase with Distinct Properties from *Bacillus thuringiensis*. *Lett. Appl. Microbiol.* **2004**, *39*, 407–412. https://doi.org/10.1111/j.1472-765X.2004.01599.x.

(7) Son, H. F.; Lee, S. H.; Lee, S. H.; Kim, H.; Hong, H.; Lee, U. J.; Lee, P. G.; Kim, B. G.; Kim, K. J. Structural Basis for Highly Efficient Production of Catechol Derivatives at Acidic pH by Tyrosinase from *Burkholderia thailandensis*. *ACS Catal.* **2018**, *8*, 10375–10382. https://doi.org/10.1021/acscatal.8b02635.

(8) McMahon, A. M.; Doyle, E. M.; Brooks, S.; O'Connor, K. E. Biochemical

Characterisation of the Coexisting Tyrosinase and Laccase in the Soil Bacterium *Pseudomonas putida* F6. *Enzyme Microb. Technol.* **2007**, *40*, 1435–1441. https://doi.org/10.1016/j.enzmictec.2006.10.020.

(9) Hernández-Romero, D.; Solano, F.; Sanchez-Amat, A. Polyphenol Oxidase Activity Expression in *Ralstonia solanacearum*. *Appl. Environ. Microbiol.* **2005**, *71*, 6808–6815. https://doi.org/10.1128/AEM.71.11.6808-6815.2005.

(10) Cabrera-Valladares, N.; Martínez, A.; Piñero, S.; Lagunas-Muñoz, V. H.; Tinoco, R.; De Anda, R.; Vázquez-Duhalt, R.; Bolívar, F.; Gosset, G. Expression of the MelA Gene from *Rhizobium etli* CFN42 in *Escherichia coli* and Characterization of the Encoded Tyrosinase. *Enzyme Microb. Technol.* **2006**, *38*, 772–779. https://doi.org/10.1016/j.enzmictec.2005.08.004.

(11) Dolashki, A.; Gushterova, A.; Voelter, W.; Tchorbanov, B. Purification and Characterization of Tyrosinases from *Streptomyces albus*. *Z. Naturforsch. C.* **2009**, *64*, 724–732. https://doi.org/10.1515/znc-2009-9-1019.

(12) Streffer, K.; Vijgenboom, E.; Tepper, A. W. J. W.; Makower, A.; Scheller, F. W.; Canters, G. W.; Wollenberger, U. Determination of Phenolic Compounds Using Recombinant Tyrosinase from S*treptomyces antibioticus*. *Anal. Chim. Acta* **2001**, *427*, 201–210. https://doi.org/10.1016/S0003-2670(00)01040-0.

(13) Bubacco, L.; Vijgenboom, E.; Gobin, C.; Tepper, A. W. J. W.; Salgado, J.; Canters, G. W. Kinetic and Paramagnetic NMR Investigations of the Inhibition of *Streptomyces antibioticus* Tyrosinase. *J. Mol. Catal. - B Enzym.* **2000**, *8*, 27–35. https://doi.org/10.1016/S1381-1177(99)00064-8.

(14) Harir, M.; Bellahcene, M.; Baratto, M. C.; Pollini, S.; Rossolini, G. M.; Trabalzini, L.; Fatarella, E.; Pogni, R. Isolation and Characterization of a Novel Tyrosinase Produced by Sahara Soil Actinobacteria and Immobilization on Nylon Nanofiber Membranes. *J. Biotechnol.* **2018**, *265*, 54–64. https://doi.org/10.1016/j.jbiotec.2017.11.004.

(15) Lerch, K.; Ettlinger, L. Purification and Properties of a Tyrosinase from *Streptomyces glaucescens*. *Eur. J. Biochem.* **1972**, *31*, 427–437. https://doi.org/10.1111/j.1432-1033.1972.tb02549.x.

(16) Guo, J.; Rao, Z.; Yang, T.; Man, Z.; Xu, M.; Zhang, X.; Yang, S. T. Cloning and

Identification of a Novel Tyrosinase and Its Overexpression in *Streptomyces kathirae* SC-1 for Enhancing Melanin Production. *FEMS Microbiol. Lett.* **2015**, *362*, fnv041. https://doi.org/10.1093/femsle/fnv041.

(17) Philipp, S.; Held, T.; Kutzner, H. J. Purification and Characterization of the Tyrosinase of *Streptomyces michiganensis* DSM 40015. *J. Basic Microbiol.* **1991**, *31*, 293–300. https://doi.org/10.1002/jobm.3620310412.

(18) Ito, M.; Oda, K. An Organic Solvent Resistant Tyrosinase from *Streptomyces sp.* REN-21: Purification and Characterization. *Bioscience, Biotechnology and Biochemistry*. **2000**, *64*, 261–267. https://doi.org/10.1271/bbb.64.261.

(19) Panis, F.; Krachler, R. F.; Krachler, R.; Rompel, A. Expression, Purification, and Characterization of a Well-Adapted Tyrosinase from Peatlands Identified by Partial Community Analysis. *Environ. Sci. Technol.* **2021**, *55*, 11445–11454. https://doi.org/10.1021/acs.est.1c02514.

(20) Kong, K.-H.; Hong, M.-P.; Choi, S.-S.; Kim, Y.-T.; Cho, S.-H. Purification and Characterization of a Highly Stable Tyrosinase from *Thermomicrobium roseum*. *Biotechnol. Appl. Biochem.* **2000**, *31*, 113–118. https://doi.org/10.1042/ba19990096.

(21) Dalcin Martins, P.; Danczak, R. E.; Roux, S.; Frank, J.; Borton, M. A.; Wolfe, R. A.; Burris, M. N.; Wilkins, M. J. Viral and Metabolic Controls on High Rates of Microbial Sulfur and Carbon Cycling in Wetland Ecosystems. *Microbiome* **2018**, *6*, 138. https://doi.org/10.1186/s40168-018-0522-4.

(22) Pankratov, T. A.; Serkebaeva, Y. M.; Kulichevskaya, I. S.; Liesack, W.; Dedysh, S. N. Substrate-Induced Growth and Isolation of Acidobacteria from Acidic *Sphagnum* Peat. *ISME J.* **2008**, *2*, 551–560. https://doi.org/10.1038/ismej.2008.7.

(23) Hausmann, B.; Pelikan, C.; Herbold, C. W.; Köstlbacher, S.; Albertsen, M.; Eichorst, S. A.; Glavina Del Rio, T.; Huemer, M.; Nielsen, P. H.; Rattei, T.; Stingl, U.; Tringe, S. G.; Trojan, D.; Wentrup, C.; Woebken, D.; Pester, M.; Loy, A. Peatland Acidobacteria with a Dissimilatory Sulfur Metabolism. *ISME J.* **2018**, *12*, 1729–1742. https://doi.org/10.1038/s41396-018-0077-1.

(24) Paul, S.; Küsel, K.; Alewell, C. Reduction Processes in Forest Wetlands:

Tracking down Heterogeneity of Source/Sink Functions with a Combination of Methods. *Soil Biol. Biochem.* **2006**, *38*, 1028–1039. https://doi.org/10.1016/j.soilbio.2005.09.001.

(25) Hausmann, B.; Knorr, K. H.; Schreck, K.; Tringe, S. G.; Glavina Del Rio, T.; Loy, A.; Pester, M. Consortia of Low-Abundance Bacteria Drive Sulfate Reduction-Dependent Degradation of Fermentation Products in Peat Soil Microcosms. *ISME J.* **2016**, *10*, 2365–2375. https://doi.org/10.1038/ismej.2016.42.

(26) Parte, A. C.; Sardà Carbasse, J.; Meier-Kolthoff, J. P.; Reimer, L. C.; Göker, M. List of Prokaryotic Names with Standing in Nomenclature (LPSN) Moves to the DSMZ. *Int. J. Syst. Evol. Microbiol.* **2020**, *70*, 5607–5612. https://doi.org/10.1099/ijsem.0.004332.

(27) Pankratov, T. A.; Dedysh, S. N. *Granulicella paludicola gen. nov., sp. nov.*, *Granulicella pectinivorans sp. nov.*, *Granulicella aggregans sp. nov*. and *Granulicella rosea sp. nov.*, Acidophilic, Polymer-Degrading Acidobacteria from *Sphagnum* Peat Bogs. *Int. J. Syst. Evol. Microbiol.* **2010**, *60*, 2951–2959. https://doi.org/10.1099/ijs.0.021824-0.

(28) Takeuchi, M.; Hatano, K. A*gromyces luteolus sp. nov.*, *Agromyces rhizospherae sp. nov*. and *Agromyces bracchium sp. nov.*, from the Mangrove Rhizosphere. *Int. J. Syst. Evol. Microbiol.* **2001**, *51*, 1529–1537. https://doi.org/10.1099/00207713-51-4-1529.

(29) Xie, Y.; Zhou, S.; Xu, Y.; Wu, W.; Xia, W.; Zhang, R.; Huang, D.; Huang, X. *Gordonia mangrovi sp. nov.*, a Novel Actinobacterium Isolated from Mangrove Soil in Hainan. *Int. J. Syst. Evol. Microbiol.* **2020**, *70*, 4537–4543. https://doi.org/10.1099/ijsem.0.004310.

(30) Li, F.; Liu, S.; Lu, Q.; Zheng, H.; Osterman, I. A.; Lukyanov, D. A.; Sergiev, P. V.; Dontsova, O. A.; Liu, S.; Ye, J.; Huang, D.; Sun, C. Studies on Antibacterial Activity and Diversity of Cultivable Actinobacteria Isolated from Mangrove Soil in Futian and Maoweihai of China. *eCAM.* **2019**, *2019*, 3476567. https://doi.org/10.1155/2019/3476567.

(31) Hu, D.; Chen, Y.; Sun, C.; Jin, T.; Fan, G.; Liao, Q.; Mok, K. M.; Lee, M. Y. S.

Genome Guided Investigation of Antibiotics Producing Actinomycetales Strain Isolated from a Macau Mangrove Ecosystem. *Sci. Rep.* **2018**, *8*, 14271. https://doi.org/10.1038/s41598-018-32076-z.

(32) Mangamuri, U. K.; Muvva, V.; Poda, S.; Manavathi, B.; Bhujangarao, C.; Yenamandra, V. Chemical Characterization & Bioactivity of Diketopiperazine Derivatives from the Mangrove Derived *Pseudonocardia endophytica*. *Egypt. J. Aquat. Res.* **2016**, *42*, 169–175. https://doi.org/10.1016/j.ejar.2016.03.001.

(33) Suriyachadkun, C.; Chunhametha, S.; Ngaemthao, W.; Tamura, T.; Kirtikara, K.; Sanglier, J. J.; Kitpreechavanich, V. *Sphaerisporangium krabiense sp. nov.*, Isolated from Soil. *Int. J. Syst. Evol. Microbiol.* **2011**, *61*, 2890–2894. https://doi.org/10.1099/ijs.0.027151-0.

(34) Zenova, G. M.; Glushkova, N. A.; Bannikov, M. V.; Shvarov, A. P.; Pozdnyakov, A. I.; Zvyagintsev, D. G. Actinomycetal Complexes in Drained Peat Soils of the Taiga Zone upon Pyrogenic Succession. *Eurasian Soil Sci.* **2008**, *41*, 394–399. https://doi.org/10.1134/S1064229308040054.

(35) Xu, D. B.; Ye, W. W.; Han, Y.; Deng, Z. X.; Hong, K. Natural Products from Mangrove Actinomycetes. *Mar. Drugs* **2014**, *12*, 2590–2613. https://doi.org/10.3390/md12052590.

(36) Gupta, N.; Mishra, S.; Basak, U. C. Occurrence of *Streptomyces aurantiacus* in Mangroves of Bhitarkanika. *Malays. J. Microbiol.* **2007**, *3*, 7–14. https://doi.org/10.21161/mjm.00807.

(37) Gong, B.; Chen, S.; Lan, W.; Huang, Y.; Zhu, X. Antibacterial and Antitumor Potential of Actinomycetes Isolated from Mangrove Soil in the Maowei Sea of the Southern Coast of China. *Iran. J. Pharm. Res.* **2018**, *17*, 1339–1346. https://doi.org/10.22037/ijpr.2018.2280.

(38) Kannan, R. R.; Vincent, S. G. P. Molecular Characterization of Antagonistic *Streptomyces* Isolated from a Mangrove Swamp. **2011**, *3*, 237–245. https://doi.org/10.3923/ajbkr.2011.237.245.

(39) Khandelwal, K. C.; Gaur, A. C. Degradation of Humic Acids, Extracted from Manure and Soil by Some *Streptomycetes* and Fungi. *Zentralblatt für Bakteriol. Parasitenkd. Infekt. und Hyg. Zweite Abteilung* **1980**, *135*, 119–122.

https://doi.org/10.1016/s0323-6056(80)80014-0.

(40) Gomathi, A.; Gothandam, K. M. Investigation of Anti-Inflammatory and Toxicity Effects of Mangrove-Derived *Streptomyces rochei* Strain VITGAP173. *J. Cell. Biochem.* **2019**, *120*, 17080–17097. https://doi.org/10.1002/jcb.28969.

(41) Raghava Rao, K. V.; Raghava Rao, T. Molecular Characterization and Its Antioxidant Activity of a Newly Isolated *Streptomyces coelicoflavus* BC 01 from Mangrove Soil. *J. Young Pharm.* **2013**, *5*, 121–126. https://doi.org/10.1016/j.jyp.2013.10.002.

(42) Ser, H.-L.; Law, J. W.-F.; Tan, W.-S.; Yin, W.-F.; Chan, K.-G. Whole Genome Sequence of *Streptomyces colonosanans* Strain MUSC 93JT Isolated from Mangrove Forest in Malaysia. *Prog. Microbes Mol. Biol.* **2020**, *3*, 1–4. https://doi.org/10.36877/pmmb.a0000061.

(43) Maidin, M. S. T.; Safari, S.; Ghani, N. A.; Ibrahim, S. A. S.; Bakeri, S. A.; Masri, M. M. M.; Ali, S. R. A. Differences in Prokaryotic Species between Primary and Logged-over Deep Peat Forest in Sarawak, Malaysia. *J. Oil Palm Res.* **2016**, *28*, 281–295. https://doi.org/10.21894/jopr/2016.2803.05.

(44) Gui, C.; Liu, Y.; Zhou, Z.; Zhang, S.; Hu, Y.; Gu, Y. C.; Huang, H.; Ju, J. Angucycline Glycosides from Mangrove-Derived *Streptomyces diastaticus* Subsp. Scsio Gj056. *Mar. Drugs* **2018**, *16*, 185. https://doi.org/10.3390/md16060185.

(45) Ruan, C. Y.; Zhang, L.; Ye, W. W.; Xie, X. C.; Srivibool, R.; Duangmal, K.; Pathom-aree, W.; Deng, Z. X.; Hong, K. *Streptomyces ferrugineus sp. nov.*, Isolated from Mangrove Soil in Thailand. *Antonie van Leeuwenhoek, Int. J. Gen. Mol. Microbiol.* **2015**, *107*, 39–45. https://doi.org/10.1007/s10482-014-0301-6.

(46) Guan, S.; Grabley, S.; Groth, I.; Lin, W.; Christner, A.; Guo, D.; Sattler, I. Structure Determination of Germacrane-Type Sesquiterpene Alcohols from an Endophyte *Streptomyces griseus Subsp. Magn. Reson. Chem.* **2005**, *43*, 1028–1031. https://doi.org/10.1002/mrc.1710.

(47) Guan, S. H.; Sattler, I.; Lin, W. H.; Guo, D. A.; Grabley, S. P-Aminoacetophenonic Acids Produced by a Mangrove Endophyte:

Streptomyces griseus Subsp. *J. Nat. Prod.* **2005**, *68*, 1198–1200. https://doi.org/10.1021/np0500777.

(48) Jacob, N.; Prema, P. Influence of Mode of Fermentation on Production of Polygalacturonase by a Novel Strain of *Streptomyces lydicus*. *Food Technol. Biotechnol.* **2006**, *44*, 263–267.

(49) Ser, H. L.; Palanisamy, U. D.; Yin, W. F.; Chan, K. G.; Goh, B. H.; Lee, L. H. *Streptomyces malaysiense sp. nov*.: A Novel Malaysian Mangrove Soil Actinobacterium with Antioxidative Activity and Cytotoxic Potential against Human Cancer Cell Lines. *Sci. Rep.* **2016**, *6*, 24247. https://doi.org/10.1038/srep24247.

(50) Ser, H. L.; Tan, W. S.; Ab Mutalib, N. S.; Yin, W. F.; Chan, K. G.; Goh, B. H.; Lee, L. H. Genome Sequence of *Streptomyces mangrovisoli* MUSC 149T Isolated from Intertidal Sediments. *Brazilian J. Microbiol.* **2018**, *49*, 13–15. https://doi.org/10.1016/j.bjm.2017.01.013.

(51) Law, J. W. F.; Ser, H. L.; Ab Mutalib, N. S.; Saokaew, S.; Duangjai, A.; Khan, T. M.; Chan, K. G.; Goh, B. H.; Lee, L. H. *Streptomyces monashensis sp. nov.*, a Novel Mangrove Soil Actinobacterium from East Malaysia with Antioxidative Potential. *Sci. Rep.* **2019**, *9*, 3056. https://doi.org/10.1038/s41598-019-39592-6.

(52) Chen, C.; Ye, Y.; Wang, R.; Zhang, Y.; Wu, C.; Debnath, S. C.; Ma, Z.; Wang, J.; Wu, M. *Streptomyces nigra sp. nov*. is a Novel Actinobacterium Isolated from Mangrove Soil and Exerts a Potent Antitumor Activity *in Vitro*. *Front. Microbiol.* **2018**, *9*, 1587. https://doi.org/10.3389/fmicb.2018.01587.

(53) Muthusamy, S.; Selvan, S. T.; Arunachalam, P.; Grasian, I. Bioconversion and Bioethanol Production from Agro-Residues through Fermentation Process Using Mangrove-Associated Actinobacterium *Streptomyces olivaceus* (MSU3). *Biofuels* **2019**, *10*, 167–179. https://doi.org/10.1080/17597269.2017.1309853.

(54) Zhao, J.; Tang, X.; Li, K.; Guo, Y.; Feng, M.; Gao, J. *Streptomyces paludis sp. nov.*, Isolated from an Alpine Wetland Soil. *Int. J. Syst. Evol. Microbiol.* **2020**, *70*, 773–778. https://doi.org/10.1099/ijsem.0.003821.

(55) Lee, L. H.; Zainal, N.; Azman, A. S.; Eng, S. K.; Ab Mutalib, N. S.; Yin, W. F.;

Chan, K. G. *Streptomyces pluripotens sp. nov.*, A Bacteriocin-Producing *Streptomycete* that Inhibits Meticillin-Resistant *Staphylococcus aureus*. *Int. J. Syst. Evol. Microbiol.* **2014**, *64*, 3297–3306. https://doi.org/10.1099/ijs.0.065045-0.

(56) Zhu, P.; Xu, Y.; Fu, J.; Liao, Y. *Streptomyces qinzhouensis sp. nov.*, a Mangrove Soil Actinobacterium. *Int. J. Syst. Evol. Microbiol.* **2020**, *70*, 1800–1804. https://doi.org/10.1099/ijsem.0.003974.

(57) Ser, H.-L.; Tan, L. T.-H.; Tan, W.-S.; Yin, W.-F.; Chan, K.-G. Whole-Genome Sequence of Bioactive *Streptomycete* Derived from Mangrove Forest in Malaysia, *Streptomyces sp.* MUSC 14. *Prog. Microbes Mol. Biol.* **2021**, *4*, a0000195. https://doi.org/10.36877/pmmb.a0000195.

(58) Kemung, H. M.; Tan, L. T. H.; Chan, K. G.; Ser, H. L.; Law, J. W. F.; Lee, L. H.; Goh, B. H. Antioxidant Activities of *Streptomyces sp*. Strain MUSC 14 from Mangrove Forest Soil in Malaysia. *Biomed Res. Int.* **2020**, *2020*, 6402607. https://doi.org/10.1155/2020/6402607.

(59) Lee, L. H.; Zainal, N.; Azman, A. S.; Eng, S. K.; Goh, B. H.; Yin, W. F.; Ab Mutalib, N. S.; Chan, K. G. Diversity and Antimicrobial Activities of Actinobacteria Isolated from Tropical Mangrove Sediments in Malaysia. *Sci. World J.* **2014**, *2014*, 698178. https://doi.org/10.1155/2014/698178.

(60) Bayot-Custodio, A. N.; Alcantara, E. P.; Zulaybar, T. O. Draft Genome Sequence of Insecticidal *Streptomyces sp.* Strain PCS3-D2, Isolated from Mangrove Soil in Philippines. *Genome Announc.* **2014**, *2*, 2–3. https://doi.org/10.1128/genomeA.00448-14.

(61) Zhou, S.; Song, L.; Masschelein, J.; Sumang, F. A. M.; Papa, I. A.; Zulaybar, T. O.; Custodio, A. B.; Zabala, D.; Alcantara, E. P.; De Los Santos, E. L. C.; Challis, G. L. Pentamycin Biosynthesis in Philippine *Streptomyces sp.* S816: Cytochrome P450-Catalyzed Installation of the C-14 Hydroxyl Group. *ACS Chem. Biol.* **2019**, *14*, 1305–1309. https://doi.org/10.1021/acschembio.9b00270.

(62) Li, Y.; Li, Y.; Li, Q.; Gao, J.; Wang, J.; Luo, Y.; Fan, X.; Gu, P. Biosynthetic and Antimicrobial Potential of Actinobacteria Isolated from Bulrush Rhizospheres

Habitat in Zhalong Wetland, China. *Arch. Microbiol.* **2018**, *200*, 695–705. https://doi.org/10.1007/s00203-018-1474-6.

(63) Meng-xi, L. I.; Hui-bin, H.; Jie-yun, L.; Jing-xiao, C. A. O.; Zhen-wang, Z. Antibacterial Performance of a *Streptomyces spectabilis* Strain producing Metacycloprodigiosin. *Curr. Microbiol.* **2021**, *78*, 2569–2576. https://doi.org/10.1007/s00284-021-02513-w.

(64) Mishra, R. K.; Kumar Mishra, D.; Ravindra; Yadav, M. K.; Pradhan, P. K.; Swaminathan, T. R.; Sood, N. Bacterial Diversity and Antibiotic Resistance in a Wetland of Lakhimpur- Kheri, Uttar Pradesh, India. *J. Environ. Biol.* **2017**, *38*, 55–66. https://doi.org/10.22438/jeb/38/1/MS-117.

(65) Kurniawan, A.; Prihanto, A. A.; Sari, S. P.; Febriyanti, D.; Kurniawan, A.; Sambah, A. B.; Asriani, E. Isolation and Identification of Cellulolytic Bacteria from Mangrove Sediment in Bangka Island. *IOP Conf. Ser. Earth Environ. Sci.* **2018**, *137*, 012070. https://doi.org/10.1088/1755-1315/137/1/012070.

(66) Auta, H. S.; Emenike, C. U.; Fauziah, S. H. Screening of *Bacillus* Strains Isolated from Mangrove Ecosystems in Peninsular Malaysia for Microplastic Degradation. *Environ. Pollut.* **2017**, *231*, 1552–1559. https://doi.org/10.1016/j.envpol.2017.09.043.

(67) Alvarenga, N.; Birolli, W. G.; Meira, E. B.; Lucas, S. C. O.; de Matos, I. L.; Nitschke, M.; Romão, L. P. C.; Porto, A. L. M. Biotransformation and Biodegradation of Methyl Parathion by Brazilian Bacterial Strains Isolated from Mangrove Peat. *Biocatal. Agric. Biotechnol.* **2018**, *13*, 319–326. https://doi.org/10.1016/j.bcab.2017.12.015.

(68) Thenmozhi, C.; Sankar, R.; Karuppiah, V.; Sampathkumar, P. *L*-Asparaginase Production by Mangrove Derived *Bacillus Cereus* MAB5: Optimization by Response Surface Methodology. *Asian Pac. J. Trop. Med.* **2011**, *4*, 486–491. https://doi.org/10.1016/S1995-7645(11)60132-6.

(69) Chantarasiri, A. Aquatic *Bacillus cereus* JD0404 Isolated from the Muddy Sediments of Mangrove Swamps in Thailand and Characterization of Its Cellulolytic Activity. *Egypt. J. Aquat. Res.* **2015**, *41*, 257–264. https://doi.org/10.1016/j.ejar.2015.08.003.

(70) Kathiresan, K.; Selvam, M. M. Evaluation of Beneficial Bacteria from Mangrove Soil. *Bot. Mar.* **2006**, *49*, 86–88. https://doi.org/10.1515/BOT.2006.011.

(71) Mishra, R. R.; Prajapati, S.; Das, J.; Dangar, T. K.; Das, N.; Thatoi, H. Reduction of Selenite to Red Elemental Selenium by Moderately Halotolerant *Bacillus megaterium* Strains Isolated from Bhitarkanika Mangrove Soil and Characterization of Reduced Product. *Chemosphere* **2011**, *84*, 1231–1237. https://doi.org/10.1016/j.chemosphere.2011.05.025.

(72) Geetha, I.; Prabakaran, G.; Paily, K. P.; Manonmani, A. M.; Balaraman, K. Characterisation of Three Mosquitocidal *Bacillus* Strains Isolated from Mangrove Forest. *Biol. Control* **2007**, *42*, 34–40. https://doi.org/10.1016/j.biocontrol.2007.04.003.

(73) Alarfaj, A. A.; Arshad, M.; Sholkamy, E. N.; Munusamy, M. A. Extraction and Characterization of Polyhydroxybutyrates (PHB) from *Bacillus thuringiensis* KSADL127 Isolated from Mangrove Environments of Saudi Arabia. *Brazilian Arch. Biol. Technol.* **2015**, *58*, 781–788. https://doi.org/10.1590/S1516-891320150500003.

(74) Maeda, M.; Mizuki, E.; Hara, M.; Tanaka, R.; Akao, T.; Yamashita, S.; Ohba, M. Isolation of B*acillus thuringiensis* from Intertidal Brackish Sediments in Mangroves. *Microbiol. Res.* **2001**, *156*, 195–198. https://doi.org/10.1078/0944-5013-00092.

(75) Nursyirwani, N.; Samiaji, J.; Tanjung, A.; Effendi, I.; Claudia, K. M. Growth and Enzyme Production of Proteolytic Bacteria from Mangrove Sediment. *IOP Conf. Ser. Earth Environ. Sci.* **2021**, *695*, 012044. https://doi.org/10.1088/1755-1315/695/1/012044.

(76) Liu, P.; Cheng, D.; Miao, L. Characterization of Thermotolerant Chitinases Encoded by a B*revibacillus laterosporusstrain* Isolated from a Suburban Wetland. *Genes.* **2015**, *6*, 1268–1282. https://doi.org/10.3390/genes6041268.

(77) Opelt, K.; Chobot, V.; Hadacek, F.; Schönmann, S.; Eberl, L.; Berg, G. Investigations of the Structure and Function of Bacterial Communities Associated with Sphagnum Mosses. *Environ. Microbiol.* **2007**, *9*, 2795–2809. https://doi.org/10.1111/j.1462-2920.2007.01391.x.

(78) Belova, S. E.; Kulichevskaya, I. S.; Akhmet’eva, N. P.; Dedysh, S. N. Shifts in a Bacterial Community Composition of a Mesotrophic Peatland after Wildfire. *Microbiol.* **2014**, *83*, 813–819. https://doi.org/10.1134/S0026261714060022.

(79) Kulichevskaya, I. S.; Ivanova, A. A.; Naumoff, D. G.; Beletsky, A. V.; Rijpstra, W. I. C.; Sinninghe Damsté, J. S.; Mardanov, A. V.; Ravin, N. V.; Dedysh, S. N. *Frigoriglobus tundricola gen. nov., sp. nov*., a Psychrotolerant Cellulolytic Planctomycete of the Family *Gemmataceae* from a Littoral Tundra Wetland. *Syst. Appl. Microbiol.* **2020**, *43*, 126129. https://doi.org/10.1016/j.syapm.2020.126129.

(80) Kulichevskaya, I. S.; Ivanova, A. O.; Baulina, O. I.; Bodelier, P. L. E.; Sinninghe Damsté, J. S. S.; Dedysh, S. N. *Singulisphaera Acidiphila gen. nov., sp. nov.*, a Non-Filamentous, Isosphaera-like Planctomycete from Acidic Northen Wetlands. *Int. J. Syst. Evol. Microbiol.* **2008**, *58*, 1186–1193. https://doi.org/10.1099/ijs.0.65593-0.

(81) Guo, M.; Han, X.; Jin, T.; Zhou, L.; Yang, J.; Li, Z.; Chen, J.; Geng, B.; Zou, Y.; Wan, D.; Li, D.; Dai, W.; Wang, H.; Chen, Y.; Ni, P.; Fang, C.; Yang, R. Genome Sequences of Three Species in the Family *Planctomycetaceae*. *J. Bacteriol.* **2012**, *194*, 3740–3741. https://doi.org/10.1128/JB.00639-12.

(82) Reimer, L. C.; Vetcininova, A.; Carbasse, J. S.; Söhngen, C.; Gleim, D.; Ebeling, C.; Overmann, J. BacDive in 2019: Bacterial Phenotypic Data for High-Throughput Biodiversity Analysis. *Nucleic Acids Res.* **2019**, *47*, D631–D636. https://doi.org/10.1093/nar/gky879.

(83) Singh, N. K.; Khatri, I.; Subramanian, S.; Mayilraj, S. Genome Sequencing and Annotation of *Acinetobacter guillouia*e Strain MSP 4-18. *Genomics Data* **2014**, *2*, 1–3. https://doi.org/10.1016/j.gdata.2013.10.001.

(84) Grouzdev, D. S.; Tikhonova, E. N.; Kravchenko, I. K. Genome Sequences of Novel A*zospirillum sp.* Strains B21 and Sh1, Isolated from Raised *Sphagnum* Bogs, and Type Strains *Azospirillum lipoferum* 59b and *Azospirillum oryzae* COC8. *Microbiol Resour Announc.* **2019**, *8*, e01174-19. https://doi.org/10.1128/MRA.01174-19.

(85) Tikhonova, E. N.; Grouzdev, D. S.; Kravchenko, I. K. *Azospirillum palustre sp.*

nov., a Methylotrophic Nitrogen-Fixing Species Isolated from Raised Bog. *Int. J. Syst. Evol. Microbiol.* **2019**, *69*, 2787–2793. https://doi.org/10.1099/ijsem.0.003560.

(86) He, C.; Zheng, L.; Ding, J.; Gao, W.; Chi, W.; Ding, Y. Complete Genome Sequence of an N-Acyl Homoserine Lactone Producer, *Breoghania sp.* Strain L-A4, Isolated from Rhizosphere of *Phragmites australis* in a Coastal Wetland. *Microbiol Resour Announc.* **2019**, *8*, e01539-18. https://doi.org/10.1128/MRA.01539-18.

(87) Catter, K. M.; Oliveira, D. F.; Sousa, O. V.; Gonçalves, L. R. B.; Fernandes Vieira, R. H. S.; Alves, C. R. Biosurfactant Production by *Pseudomonas aeruginosa* and *Burkholderia gladioli* Isolated from Mangrove Sediments Using Alternative Substrates. *Orbital - Electron. J. Chem.* **2016**, *8*, 5. https://doi.org/10.17807/orbital.v8i5.771.

(88) Ong, K. S.; Aw, Y. K.; Lee, L. H.; Yule, C. M.; Cheow, Y. L.; Lee, S. M. *Burkholderia Paludis sp. nov.*, an Antibiotic-Siderophore Producing Novel *Burkholderia cepacia* Complex Species, Isolated from Malaysian Tropical Peat Swamp Soil. *Front. Microbiol.* **2016**, *7*, 1–14. https://doi.org/10.3389/fmicb.2016.02046.

(89) Hetz, S. A.; Poehlein, A.; Horn, M. A. Whole-Genome Sequences of Two New *Caballeronia* Strains Isolated from Cryoturbated Peat Circles of the Permafrost-Affected Eastern European Tundra. *Microbiol. Resour. Announc.* **2020**, *9*, 8–10. https://doi.org/10.1128/mra.00731-20.

(90) Blackburn, M. B.; Farrar, R. R.; Sparks, M. E.; Kuhar, D.; Mitchell, A.; Gundersen-Rindal, D. E. *Chromobacterium sphagni sp. nov.,* an Insecticidal Bacterium Isolated from *Sphagnum* Bogs. *Int. J. Syst. Evol. Microbiol.* **2017**, *67*, 3417–3422. https://doi.org/10.1099/ijsem.0.002127.

(91) Soby, S. D.; Gadagkar, S. R.; Contreras, C.; Caruso, F. L. *Chromobacterium vaccinii sp. nov.*, Isolated from Native and Cultivated Cranberry (*Vaccinium macrocarpon* Ait.) Bogs and Irrigation Ponds. *Int. J. Syst. Evol. Microbiol.* **2013**, *63*, 1840–1846. https://doi.org/10.1099/ijs.0.045161-0.

(92) Ye, Y. H.; Anwar, N.; Xamxidin, M.; Zhang, R.; Yan, C.; Nie, Y. F.; Zhao, Z.;

Sun, C.; Wu, M. Description of *Erythrobacter mangrovi sp. nov.*, an Aerobic Bacterium from Rhizosphere Soil of Mangrove Plant (*Kandelia candel*). *Antonie van Leeuwenhoek* **2020**, *113*, 1425–1435. https://doi.org/10.1007/s10482-020-01451-0.

(93) Dinesh, B.; Lau, N. S.; Furusawa, G.; Kim, S. W.; Taylor, T. D.; Foong, S. Y.; Shu-Chien, A. C. Comparative Genome Analyses of Novel Mangrovimonas-like Strains Isolated from Estuarine Mangrove Sediments Reveal Xylan and Arabinan Utilization Genes. *Mar. Genomics* **2016**, *25*, 115–121. https://doi.org/10.1016/j.margen.2015.12.006.

(94) Sam, K.-K.; Lau, N.-S.; Furusawa, G.; Amirul, A.-A. A. Draft Genome Sequence of Halophilic *Hahella sp.* Strain CCB-MM4, Isolated from Matang Mangrove Forest in Perak, Malaysia. *Am. Scoiety Microbiol.* **2017**, *5*, e01147-17. https://doi.org/https://doi.org/10.1128/genomeA.00504-17.

(95) Graef, C.; Hestnes, A. G.; Svenning, M. M.; Frenzel, P. The Active Methanotrophic Community in a Wetland from the High Arctic. *Environ. Microbiol. Rep.* **2011**, *3*, 466–472. https://doi.org/10.1111/j.1758-2229.2010.00237.x.

(96) Wartiainen, I.; Hestnes, A. G.; McDonald, I. R.; Svenning, M. M. *Methylocystis rosea sp. nov.*, a Novel Methanotrophic Bacterium from Arctic Wetland Soil, Svalbard, Norway (78° N). *Int. J. Syst. Evol. Microbiol.* **2006**, *56*, 541–547. https://doi.org/10.1099/ijs.0.63912-0.

(97) Chung, E. J.; Park, T. S.; Kim, K. H.; Jeon, C. O.; Lee, H. I.; Chang, W. S.; Aslam, Z.; Chung, Y. R. *Nitrospirillum irinus sp. nov.*, a Diazotrophic Bacterium Isolated from the Rhizosphere Soil of Iris and Emended Description of the Genus *Nitrospirillum*. *Antonie Van Leeuwenhoek* **2015**, *108*, 721–729. https://doi.org/10.1007/s10482-015-0528-x.

(98) Kang, H.; Kim, H.; Joung, Y.; Kim, K. J.; Joh, K. *Paludibacterium purpuratum sp. nov.*, Isolated from Wetland Soil. *Int. J. Syst. Evol. Microbiol.* **2016**, *66*, 2711–2716. https://doi.org/10.1099/ijsem.0.001112.

(99) Xu, X. R.; Li, H. Bin; Gu, J. D. Metabolism and Biochemical Pathway of N-Butyl Benzyl Phthalate by *Pseudomonas Fluorescens* B-1 Isolated from a Mangrove

Sediment. *Ecotoxicol. Environ. Saf.* **2007**, *68*, 379–385. https://doi.org/10.1016/j.ecoenv.2006.11.012.

(100) Parthasarathi, R.; Sivakumaar, P. K. Effect of Different Carbon Sources on the Production of Biosurfactant by *Pseudomonas fluorescens* Isolated from Mangrove Forests (Pichavaram), Tamil Nadu, India. *Glob. J. Environ. Res.* **2009**, *3*, 99–101.

(101) Zhang, C.; Zhang, Z.; Li, J.; Qin, K.; Wei, Y.; Zhang, Q.; Lin, L.; Ji, X. Complete Genome Sequence of the Lytic Cold-Active *Pseudomonas fluorescens* Bacteriophage VSW-3 from Napahai Plateau Wetland. *Virus Genes* **2017**, *53*, 146–150. https://doi.org/10.1007/s11262-016-1403-1.

(102) Ebadzadsahrai, G.; Soby, S. Draft Genome Sequence of *Pseudomonas sp.* Strain MWU12- 2323, Isolated from a Wild Cranberry Bog in Truro, Massachusetts. *Microbiol. Resour. Announc.* **2020**, *9*, e01387-19. https://doi.org/10.1128/MRA.01387-19.

(103) Kumar, P. A.; Aparna, P.; Srinivas, T. N. R.; Sasikala, C.; Ramana, C. V. *Rhodovulum kholense sp. nov. Int. J. Syst. Evol. Microbiol.* **2008**, *58*, 1723–1726. https://doi.org/10.1099/ijs.0.65620-0.

(104) Weiner, R. M.; Taylor, L. E.; Henrissat, B.; Hauser, L.; Land, M.; Coutinho, P. M.; Rancurel, C.; Saunders, E. H.; Longmire, A. G.; Zhang, H.; Bayer, E. A.; Gilbert, H. J.; Larimer, F.; Zhulin, I. B.; Ekborg, N. A.; Lamed, R.; Richardson, P. M.; Borovok, I.; Hutcheson, S. Complete Genome Sequence of the Complex Carbohydrate-Degrading Marine Bacterium, *Saccharophagus degradans* Strain 2-40T. *PLoS Genet.* **2008**, *4*, e1000087. https://doi.org/10.1371/journal.pgen.1000087.

(105) Han, K.; Li, Z. F.; Peng, R.; Zhu, L. P.; Zhou, T.; Wang, L. G.; Li, S. G.; Zhang, X. B.; Hu, W.; Wu, Z. H.; Qin, N.; Li, Y. Z. Extraordinary Expansion of a *Sorangium cellulosum* Genome from an Alkaline Milieu. *Sci. Rep.* **2013**, *3*, 3–9. https://doi.org/10.1038/srep02101.

(106) Zhang, Q.; Wang, Y.; Zhou, J. Complete Genome Sequence of *Stenotrophomonas rhizophila* KC1, a Quorum Sensing- Producing Algicidal Bacterium, Isolated from Mangrove *Kandelia Candel*. *Mol. Plant Microbe*

Interact. **2021**, *34*, 857–861. https://doi.org/10.1094/MPMI-12-20-0346-A.

(107) UniProt Consortium. UniProt: The Universal Protein Knowledgebase in 2021. *Nucleic Acids Res.* **2021**, *49*, 480–489. https://doi.org/10.1093/nar/gkaa1100.

(108) Ren, Q.; Henes, B.; Fairhead, M.; Thöny-Meyer, L. High Level Production of Tyrosinase in Recombinant *Escherichia coli*. *BMC Biotechnol.* **2013**, *13*, 18. https://doi.org/10.1186/1472-6750-13-18.

(109) Shuster Ben-Yosef, V.; Sendovski, M.; Fishman, A. Directed Evolution of Tyrosinase for Enhanced Monophenolase/Diphenolase Activity Ratio. *Enzyme Microb. Technol.* **2010**, *47*, 372–376. https://doi.org/10.1016/j.enzmictec.2010.08.008.

(110) Goldfeder, M.; Kanteev, M.; Adir, N.; Fishman, A. Influencing the Monophenolase/Diphenolase Activity Ratio in Tyrosinase. *Biochim. Biophys. Acta* **2013**, *1834*, 629–633. https://doi.org/10.1016/j.bbapap.2012.12.021.

(111) Dolashki, A.; Voelter, W.; Gushterova, A.; Van Beeumen, J.; Devreese, B.; Tchorbanov, B. Isolation and Characterization of Novel Tyrosinase from *Laceyella sacchari*. *Protein Pept. Lett.* **2012**, *19*, 538–543. https://doi.org/10.2174/092986612800191035.

(112) Nadal-Jimenez, P.; Koch, G.; Reis, C. R.; Muntendam, R.; Raj, H.; Margot Jeronimus-Stratingh, C.; Cool, R. H.; Quax, W. J. PvdP Is a Tyrosinase That Drives Maturation of the Pyoverdine Chromophore in *Pseudomonas aeruginosa*. *J. Bacteriol.* **2014**, *196*, 2681–2690. https://doi.org/10.1128/JB.01376-13.

(113) Molloy, S.; Nikodinovic-Runic, J.; Martin, L. B.; Hartmann, H.; Solano, F.; Decker, H.; O'Connor, K. E. Engineering of a Bacterial Tyrosinase for Improved Catalytic Efficiency towards D-Tyrosine Using Random and Site Directed Mutagenesis Approaches. *Biotechnol. Bioeng.* **2013**, *110*, 1849–1857. https://doi.org/10.1002/bit.24859.

(114) Lee, N.; Lee, S. H.; Baek, K.; Kim, B. G. Heterologous Expression of Tyrosinase (MelC2) from *Streptomyces avermitilis* MA4680 in *E. coli* and Its Application for Ortho-Hydroxylation of Resveratrol to Produce Piceatannol. *Appl. Microbiol. Biotechnol.* **2015**, *99*, 7915–7924.

https://doi.org/10.1007/s00253-015-6691-1.

(115) Kohashi, P. Y.; Kumagai, T.; Matoba, Y.; Yamamoto, A.; Maruyama, M.; Sugiyama, M. An Efficient Method for the Overexpression and Purification of Active Tyrosinase from *Streptomyces castaneoglobisporus*. *Protein Expr. Purif.* **2004**, *34*, 202–207. https://doi.org/10.1016/j.pep.2003.11.015.

(116) Ser, H. L.; Palanisamy, U. D.; Yin, W. F.; Abd Malek, S. N.; Chan, K. G.; Goh, B. H.; Lee, L. H. Presence of Antioxidative Agent, Pyrrolo[1,2-a]Pyrazine-1,4-Dione, Hexahydro- in Newly Isolated *Streptomyces mangrovisoli sp. nov. Front. Microbiol.* **2015**, *6*, 854. https://doi.org/10.3389/fmicb.2015.00854.

(117) Sendovski, M.; Kanteev, M.; Ben-Yosef, V. S.; Adir, N.; Fishman, A. First Structures of an Active Bacterial Tyrosinase Reveal Copper Plasticity. *J. Mol. Biol.* **2011**, *405*, 227–237. https://doi.org/10.1016/j.jmb.2010.10.048.

(118) Matoba, Y.; Kumagai, T.; Yamamoto, A.; Yoshitsu, H.; Sugiyama, M. Crystallographic Evidence That the Dinuclear Copper Center of Tyrosinase Is Flexible during Catalysis. *J. Biol. Chem.* **2006**, *281*, 8981–8990. https://doi.org/10.1074/jbc.M509785200.

(119) Kampatsikas, I.; Rompel, A. Similar but Still Different: Which Amino Acid Residues Are Responsible for Varying Activities in Type-III Copper Enzymes? *ChemBioChem* **2021**, *22*, 1161–1175. https://doi.org/10.1002/cbic.202000647.

(120) Jia, W.; Wang, Q.; Fan, X.; Dong, A.; Yu, Y.; Wang, P. Laccase-Mediated *in Situ* Oxidation of Dopa for Bio-Inspired Coloration of Silk Fabric. *RSC Adv.* **2017**, *7*, 12977–12983. https://doi.org/10.1039/c6ra25533g.

(121) Puiu, M.; Babaligea, I.; Olmazu, C.; Răducan, A.; Oancea, D. Peroxidase-Mediated Oxidation of *L*-Dopa: A Kinetic Approach. *Biochem. Eng. J.* **2010**, *52*, 248–254. https://doi.org/10.1016/j.bej.2010.08.017.

(122) Ziegenhagen, D.; Hofrichter, M. Degradation of Humic Acids by Manganese Peroxidase from the White-Rot Fungus *Clitocybula dusenii*. *J. Basic Microbiol.* **1998**, *38*, 289–299. https://doi.org/10.1002/(SICI)1521-4028(199809)38:4<289::AID-JOBM289>3.0.CO;2-H

(123) Zavarzina, A. G.; Leontievsky, A. A.; Golovleva, L. A.; Trofimov, S. Y. Biotransformation of Soil Humic Acids by Blue Laccase of *Panus tigrinus* 8/18:

An *in Vitro* Study. *Soil Biol. Biochem.* **2004**, *36*, 359–369. https://doi.org/10.1016/j.soilbio.2003.10.010.

(124) Ramsden, C. A.; Riley, P. A. Tyrosinase: The Four Oxidation States of the Active Site and Their Relevance to Enzymatic Activation, Oxidation and Inactivation. *Bioorganic Med. Chem.* **2014**, *22*, 2388–2395. https://doi.org/10.1016/j.bmc.2014.02.048.

(125) Muñoz, J. L.; García-Molina, F.; Varón, R.; Rodriguez-Lopez, J. N.; García-Cánovas, F.; Tudela, J. Calculating Molar Absorptivities for Quinones: Application to the Measurement of Tyrosinase Activity. *Anal. Biochem.* **2006**, *351*, 128–138. https://doi.org/10.1016/j.ab.2006.01.011.
